# Synthesis and Biological Evaluation of Marine-Inspired Benzothiazole Derivatives as Retinoid X Receptor-α Antagonists with Anti-Cancer Activities

**DOI:** 10.3390/md23090368

**Published:** 2025-09-21

**Authors:** Yingting Lin, Ming Peng, Renjing Yang, Guanghui Wang, Junjie Chen, Rong Ding, Cuiling Sun, Wenjing Tian, Haifeng Chen

**Affiliations:** Fujian Provincial Key Laboratory of Innovative Drug Target, School of Pharmaceutical Sciences, Xiamen University, Xiamen 361005, China; yingting301@163.com (Y.L.); pengming.sz@foxmail.com (M.P.); 32320230157401@stu.xmu.edu.cn (R.Y.); guanghui@xmu.edu.cn (G.W.); chenjunjie@xmu.edu.cn (J.C.); dingrong@xmu.edu.cn (R.D.); cuiling@xmu.edu.cn (C.S.)

**Keywords:** benzothiazole derivatives, RXRα, structure–activity relationship, anti-cancer activity, mechanism

## Abstract

Retinoid X receptor α (RXRα) plays a vital role in multiple biological and pathological processes and represents a promising therapeutic target for anti-tumor drug design. Inspired by the marine-derived RXRα antagonist meroterpenthiazole A, 21 undescribed benzothiazole derivatives were designed and synthesized. The inhibitory effects of 21 derivatives on RXRα transactivation and their anti-tumor activities against MDA-MB-231 cells were evaluated. Compounds **4a**–**4h**, **6a**–**6b**, **7c**–**7f**, and **7h**–**7i** inhibited 9-*cis*-retinoic acid-induced RXRα transactivation, while compounds **3b**, **4f**–**4h**, **7a**, **7c**, **7f**, and **7h**–**7i** exhibited inhibitory effects on the proliferation of MDA-MB-231 cells. Meanwhile, the structure–activity relationships governing both the RXRα antagonist effects and the anti-proliferative activities against MDA-MB-231 cells were discussed. Compound **7i** exhibited the most potent inhibitory effects on the proliferation of MDA-MB-231 cells with an IC_50_ value of 16.5 μM. Further mechanism studies revealed that compound **7i** induced G2/M phase arrest in MDA-MB-231 cells, accompanied by dose-dependent downregulation of Cyclin B1 and CDK1 protein expression. However, these effects were abolished in RXRα-knockout MDA-MB-231 cells, indicating that the anti-proliferative and cell cycle arrest activities of **7i** were RXRα-dependent. Cellular Thermal Shift Assay (CETSA) and molecular docking studies further confirmed that **7i** directly bound to RXRα, thereby mediating its anti-cancer efficacy.

## 1. Introduction

Retinoid X receptor-α(RXRα), a unique member of the nuclear receptor (NR) superfamily, plays a critical role in many biological and pathological processes [1]. Dysfunctions of RXRα appear in numerous diseases, especially cancer [2,3]. The RXRα ligand-binding domain (LBD) contains a structurally defined ligand-binding pocket (LBP) that accommodates small-molecule ligands. In general, the reported RXRα-LBD ligands feature three conserved structural elements: (i) a polar head group (typically a carboxyl moiety), (ii) a conjugated polyene linker, and (iii) a hydrophobic terminal tail. Ref. [3] Bexarotene (Targretin), an FDA-approved RXRα ligand, has been clinically approved for the treatment of human cutaneous T-cell lymphoma. K-8008, as a sulindac-derived RXRα ligand, could inhibit the 9-cis-retinoic acid (9-cis-RA)-induced transactivation of RXRα and exert potent anti-cancer activity in breast cancer cells and animal models [4]. XS-060 and its derivative BPA-B9 are RXRα antagonists that could target RXRα and significantly induce RXRα-dependent mitotic arrest [5,6]. RXRα small-molecule modulators have shown great potential as anti-cancer agents and offer new approaches to tumor therapy [7].

As an important category of sulfur-containing nitrogen heterocyclic structures, benzothiazole-containing compounds have received extensive attention in the field of medicinal chemistry due to their strong structural modifiability, diverse biological activities, and good potential for drug development [8]. Benzothiazole derivates have shown broad application prospects in various fields such as antibacterial [9], antiviral [10], anti-tumor [11], anti-inflammatory [12], and neurodegenerative disease treatment [13]. Our previous research found that the benzothiazole-containing compound meroterpenthiazole A derived from the marine fungus *Penicillium allii-sativi* could bind to RXRα (*K*_D_ = 12.3 μM) and inhibit the transactivation of RXRα [14]. However, its anti-tumor activity was not significant. Its structure still needs further optimization to enhance the anti-tumor activity.

The interaction between meroterpenthiazole A and RXRα is primarily mediated through its benzothiazole core scaffold [14]. Accordingly, our structural optimization strategy retained this central benzothiazole moiety while introducing diverse side chain modifications at its accessible sites. In contrast with the structurally complex natural product meroterpenthiazole A, the designed analogs maintain the essential pharmacophoric elements within a simplified molecular framework. This approach facilitates the efficient development of lead compounds with improved biological activity. In this study, we designed and synthesized a series of benzothiazole derivates inspired by the marine-derived natural compound meroterpenthiazole A and evaluated their anti-cancer and RXRα-modulating activities.

## 2. Results and Discussion

### 2.1. Synthesis of Benzothiazole Derivatives

**Scheme 1:**
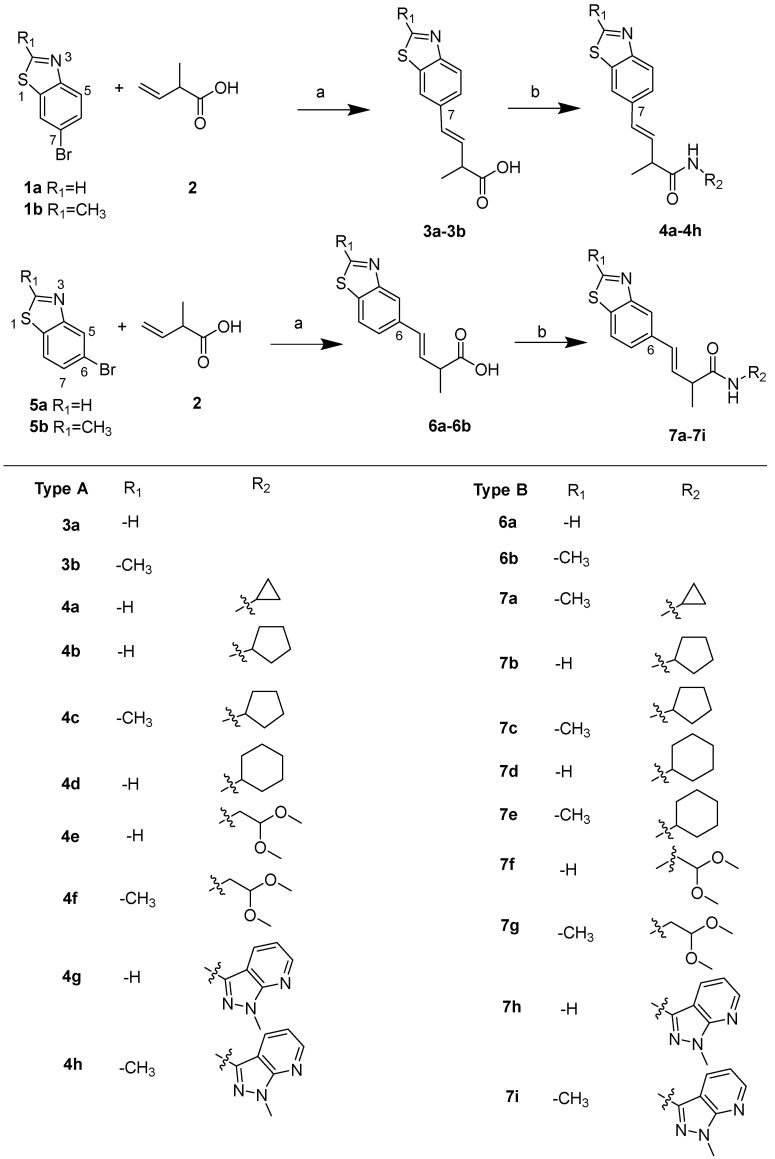
Synthesis of 21 benzothiazole derivatives, reaction condition: (a) tri-ortho-tolylphosphine, Pd (OAc)_2_, TEA (triethylamine), 1, 4-dioxane/water = 3:1 (*v*/*v*), RT 24h; (b) NMI, TCFH, MeCN, H_2_N-R_3_, RT 20 h.

Two series of benzothiazole derivatives (A and B) were synthesized as depicted in Scheme 1. Firstly, commercially available 7-bromo-1,3-benzothiazole (**1a**) or 7-bromo-2-methylbenthiazole (**1b**) reacted with 2-methylbut-3-enoic acid (**2**) at room temperature for 24 h to afford **3a**–**3b**. This process involves the Mizoroki–Heck reaction performed using Pd (OAc)_2_ as the catalyst, tri-ortho-tolylphosphine as the stabilizing ligand, triethylamine (TEA, 0.1 equiv) as the base to facilitate the palladium catalytic cycle, and a 3:1 (*v*/*v*) mixture of 1,4-dioxane/water as the solvent system. Following the above reaction conditions, compounds **6a** and **6b** were synthesized from 6-bromo-1,3-benzothiazole (**5a**) and 6-bromo-2-methylbenzothiazole (**5b**), respectively, using 2-methylbut-3-enoic acid (**2**) as a common substrate. Further amidations were conducted at the carboxyl group on the benzothiazole side chain using acetonitrile as the solvent. The reactions were carried out with N-methylimidazole (NMI) and N, N, N′, N′-tetramethylchloroformamidinium hexafluorophosphate (TCFH) as activating agents, in the presence of various substituted amines.

After stirring at room temperature for 20 h, the target compounds **4a**–**4h** and **7a**–**7i** were obtained. Compounds **3a**–**3b** and **4a**–**4h** are type A benzothiazole derivatives with the side chain substituted at C-7, while compounds **6a**–**6b** and **7a**–**7i** are type B benzothiazole derivatives with the side chain substituted at C-6.

**Figure 1:**
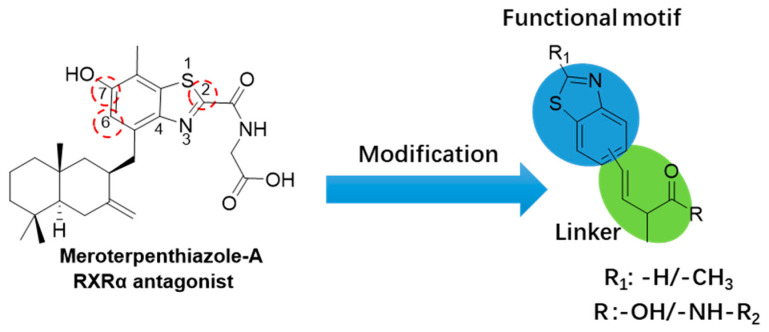
Modification sites on the benzothiazole core of the meroterpenthiazole A.

Twenty-one novel benzothiazole analogs were designed and synthesized based on the scaffold of meroterpenthiazole A, a benzothiazole-containing meroterpenoid isolated from a marine-derived fungus. As shown in Figure 1, the benzothiazole core can be selectively methylated or hydrogenated at C-2 to modulate its lipophilicity profile. Concurrently, the installation of an α, β-unsaturated system in the carboxylic acid side chain extends π-conjugation between the heterocyclic core and terminal carboxylate. Then, functionalization of the carboxyl terminus via amidation, coupled with the introduction of conjugated groups, extends the molecular framework. The above synthetic strategies were used to optimize the interaction and binding affinity to the RXRα.

Scheme 1 outlines the synthetic procedure of the designed and synthesized benzothiazole derivatives (**3a**–**3b**, **4a**–**4h**, **6a**–**6b**, and **7a**–**7i**). All 21 compounds are novel synthesized benzothiazole derivatives. All the synthesized compounds were elucidated by ^1^H NMR (600 MHz), ^13^C NMR (150 MHz), and HRMS (ESI) spectra.

### 2.2. Biological Evaluation and SAR Analysis of Benzothiazole Derivatives

#### 2.2.1. RXRα Transcriptional Activity

**Figure 2:**
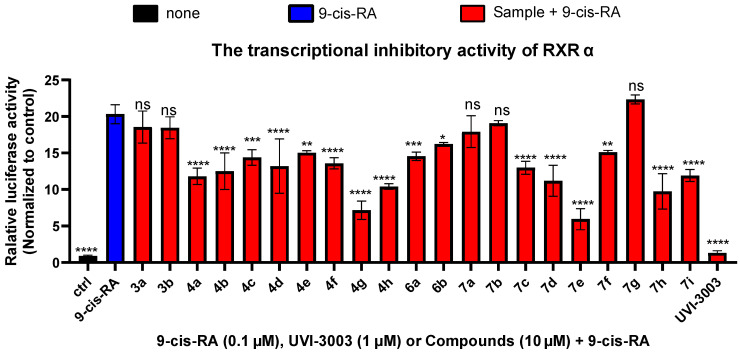
The RXRα transcriptional inhibitory activity of 21 benzothiazole derivatives. * indicates a statistically significant difference compared to the 9-cis-RA group, *p* < 0.05, *p* < 0.01 (**), *p* < 0.001 (***), *p* < 0.0001 (****).

Previously research found that a marine-derived benzothiazole-containing meroterpenthiazole A could bind to RXRα-LBD and inhibit 9-cis-RA-induced RXRα transactivation. Thus, the inhibitory activities of 21 synthetic benzothiazole derivates toward the transcription of RXRα were evaluated in HEK293T cells with a Gal4-RXRαLBD chimera and Gal4 reporter system by a dual-luciferase reporter assay. As expected, 9-cis-RA strongly activated the RXRα transcription and the UVI-3003 as a positive control strongly inhibited this activation, thereby validating the experimental system. As presented in Figure 2, compounds **4a**–**4h**, **6a**–**6b**, **7c**–**7f**, and **7h**–**7i** showed antagonist effects on the 9-cis-RA-induced RXRα transactivation.

**Figure 3:**
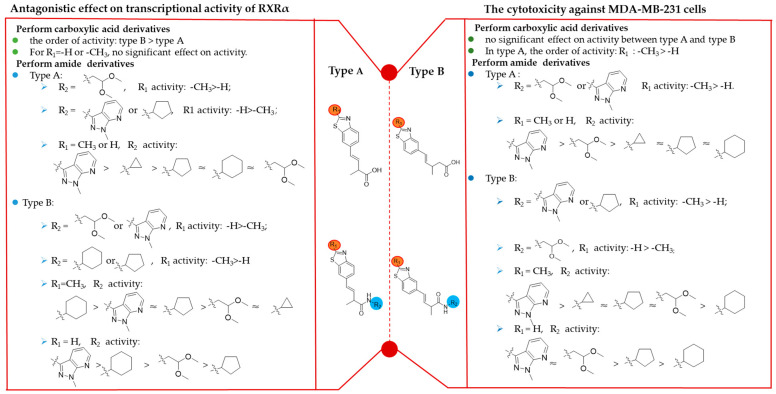
The SAR summary of benzothiazole derivatives.

The structure–activity relationship (SAR) of synthetic benzothiazole derivatives in relation to their RXRα transactivation-inhibitory activity was preliminarily discussed. As depicted in Figure 3, for the carboxylic acid derivatives, type B compounds (**6a**–**6b**) with a substituent at C-6 in the benzothiazole core exhibit better RXRα transcriptional inhibitory activities than those of type A compounds with a substituent at C-7 (**3a**–**3b**). However, no significant differences were observed when R_1_ was CH_3_ or H.

For derivatives bearing an amide moiety at the terminal position of the benzothiazole side chain, the structure–activity relationship (SAR) was illustrated as follows: For the type A compounds, the inhibitory activity was influenced by the R_1_ and R_2_ substituents. When R_2_ was 1,1-dimethoxyethyl, compound **4f** (R_1_ = CH_3_) exhibited stronger activity than **4e** (R_1_ = H). Conversely, when R_2_ was 1-methyl-1H-pyrazolo[3,4-b] pyridyl, compounds bearing R_1_ = H (**4g** and **7h**) showed higher activity than those with R_1_ = CH_3_. When R_2_ was cyclopentyl, the inhibitory activity of compound **4c** (R_1_ = CH_3_) was lower than that of **4b** (R_1_ = H). Regardless of R_1_ (CH_3_ or H), the activity order of R_2_ was 1-methyl-1H-pyrazolo [3,4-b] pyridyl > cyclopropyl > cyclohexyl ≈ cyclopentyl ≈ 1,1-dimethoxyethyl (as observed in compounds **4a**–**4h**).

For type B compounds, the SAR were discussed as follows: When R_2_ was 1,1-dimethoxyethyl or 1-methyl-1H-pyrazolo[3,4-b] pyridy, compounds with R_1_ = H were more potent than those with R_1_ = CH_3_ (i.e., **7f** > **7i**, **7h** > **7i**). When R_2_ was cyclohexyl or cyclopentyl, the CH_3_ substitution at R_1_ resulted in more potent RXRα transcriptional inhibition activity compared to the compounds with R_1_ = H (i.e., **7c** > **7b**, **7e** > **7d**). When R_1_ = CH_3_, the activity order of R_2_ was cyclohexyl > cyclopentyl ≈ 1-methyl-1H-pyrazolo[3,4-b] pyridyl > cyclopropyl ≈ 1,1-dimethoxyethyl (inferred from **7e** > **7c** ≈ **7i** > **7a** ≈ **7g**). However, when R_1_ = H, the activity order of R_2_ was 1-methyl-1H-pyrazolo[3,4-b] pyridyl > cyclohexyl > 1,1-dimethoxyethyl > cyclopentyl (based on **7h** > **7d** > **7f** > **7b**).

#### 2.2.2. In Vitro Anti-Cancer Activity

**Figure 4:**
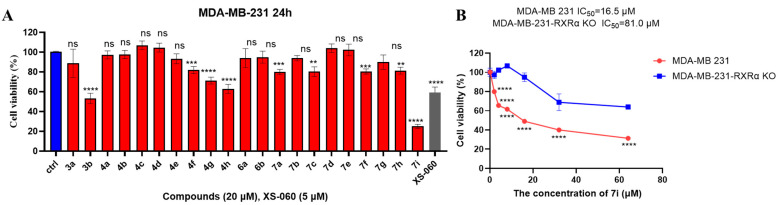
The inhibitory activities of 21 benzothiazole derivatives against MDA-MB 231 cells. (**A**) The cytotoxicity of 21 benzothiazole derivatives at a concentration of 20 μM. XS-060 (5 μM) was the positive control. (**B**) The IC_50_ curves of compound **7i** against wild-type and RXRα-knocked-out (RXRα KO) MDA-MB 231 cells. *p* < 0.01 (**), *p* < 0.001 (***), *p* < 0.0001 (****). ns, not significant.

Currently, the treatment of triple-negative breast cancer (TNBC) primarily relies on surgery, and there are no effective therapeutic drugs available. RXRα antagonists have demonstrated potential against TNBC in both in vitro and in vivo experiments, such as XS-060 and its derivative BPA-B9 [5]. The synthesized benzothiazole derivatives were also screened for in vitro cytotoxicity against MDA-MB-231 cells by an MTT assay. As shown in Figure 4A, **3b**, **4f**–**4h**, **7a**, **7c**, **7f**, and **7h**–**7i** inhibited the proliferation of MDA-MB-231 cells at a concentration of 20 μM (Figure 4A). Compound **7i** exhibited the most significant inhibitory effect with the IC_50_ value of 16.5 μM. However, the inhibitory activity of **7i** was significantly reduced by RXRα-knockout MDA-MB-231 cells with IC_50_ = 81.0 μM (Figure 4B), which indicated that the anti-cancer activity of **7i** against MDA-MB-231 cells was RXRα-dependent.

The SAR underlying the cytotoxic activity of the synthesized compounds was evaluated, and the key findings are summarized below. As shown in Figure 3, for the carboxylic acid derivatives, no significant difference in activity was observed between type A and type B compounds. However, type A compounds bearing a methyl group at C-2 exhibited stronger inhibitory activities against MDA-MB-231 cells than those of type B, as evidenced by the superior potency of compound **3b** over **6b**.

For derivatives featuring a terminal amide substitution on the benzothiazole side chain, no significant difference in activity was observed between type A and type B compounds. For the type A series of compounds, when R_2_ is 1,1-dimethoxyethyl or 1-methyl-1H-pyrazolo[3,4-b] pyridyl, the inhibitory activities of compounds **4f** and **4h** with R_1_ = CH_3_ are more potent than compounds **4e** and **4g** with R_1_ = H. When R_1_ is CH_3_ or H, the activity order of R_2_ is 1-methyl-1H-pyrazolo[3,4-b] pyridyl > 1,1-dimethoxyethyl > cyclohexyl ≈ cyclopentyl ≈ cyclopropyl, as evidenced by compounds **4a**–**4h**.

For the type B series of compounds, compound **7f** with R_1_ = H is more potent than compound **7g** with R_1_ = CH_3_. When R_2_ is 1-methyl-1H-pyrazolo[3,4-b] pyridyl or cyclopentyl, the inhibitory activity of compounds **7c** and **7i** with R_1_ = CH_3_ is greater than those of R_1_ = H. When R_2_ is cyclopentyl or 1-methyl-1H-pyrazolo[3,4-b] pyridyl, compounds with R_1_ = CH_3_ have more potent cytotoxicity against MDA-MB 231 cells than compounds with R_1_ = H, as demonstrated by the activities of **7b**–**7c** and **7h**–**7i**. When R_2_ is 1,1-dimethoxyethyl, compound **7f** with R_1_ = H have more potent cytotoxicity than **7g** with R_1_ = CH_3_. When R_1_ is CH_3_, the activity order of R_2_ substituent is 1-methyl-1H-pyrazolo[3,4-b] pyridyl > cyclopropyl ≈ cyclopentyl > 1,1-dimethoxyethyl ≈ cyclohexyl, as demonstrated by compounds **7i** > **7a** ≈ **7c** > **7g** ≈ **7e**. When R_1_ is H, the activity order of the R_2_ substituent is 1-methyl-1H-pyrazolo[3,4-b] pyridyl ≈1,1-dimethoxyethyl > cyclohexyl ≈ cyclopentyl, deduced by compounds **7f** ≈ **7h** > **7d** ≈ **7b**.

### 2.3. Compound ***7i*** Inhibits the Proliferation of MDA-MB-231 by Inducing Cell Cycle Arrest at the G2/M Phase

**Figure 5:**
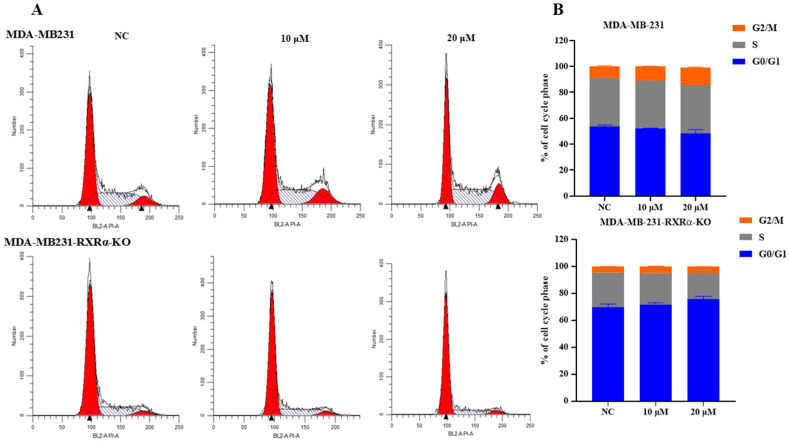
Compound **7i** caused G2/M phase arrest in MDA-MB-231 cells. (**A**) MDA-MB-231 cells and RXRα-knocked-out MDA-MB-231 cells were treated with **7i** at the concentrations of 10 μM and 20 μM for 48 h and then analyzed by flow cytometry. (**B**) Statistical analysis of relative area covered by colonies in MDA-MB-231-cells and RXRα-KO MDA-MB-231 cells.

Based on the MTT cytotoxicity results, we aimed to investigate the potential mechanisms by which **7i** inhibits MDA-MB-231 cells. Studies have demonstrated that the RXRα antagonist XS-060 and its derivates exert anti-proliferative activity against MDA-MB-231 cells by inducing G2/M phase arrest [5]. In our work, after treating MDA-MB-231 cells with **7i** for 48 h, cell cycle distribution was analyzed. As a result (Figure 5A,B), **7i** induced G2/M phase arrest in wild-type MDA-MB-231 cells at a concentration of 20 μM, with a statistically significant difference compared to the control group (*p* < 0.05). However, this G2/M phase arrest was abrogated when RXRα was knocked out, even in the presence of **7i**. Specifically, in wild-type MDA-MB-231 cells, **7i** treatment led to a significant increase in the proportion of G2/M phase cells, indicating that compound **7i** can regulate the cell cycle and induce G2/M phase arrest to inhibit the proliferation of MDA-MB-231 cells. In contrast, a significant decrease in the proportion of G2/M phase cells was observed in RXRα-knocked-out MDA-MB-231-cells, suggesting that the induction of G2/M phase arrest by **7i** was dependent on RXRα.

### 2.4. Detection of G2/M Phase Protein-Related Expression Levels by Western Blot

**Figure 6:**
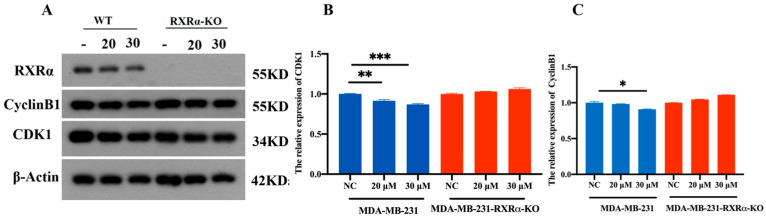
The effects of **7i** on the expression levels of Cyclin B1 and CDK1, detected by Western blot. (**A**) MDA-MB 231 cells and RXRα-KO MDA-MB 231 cells were treated with **7i** (20 μM and 30 μM) and 0.1% DMSO (NC group) for 36 h and analyzed by immunoblotting. (**B**) The grayscale analysis bar chart of CDK1 protein expression in MDA-MB 231 cells and RXRα KO MDA-MB 231 cells. (**C**) The grayscale analysis bar chart of Cyclin B1 protein expression in MDA-MB 231 cells and RXRα KO MDA-MB-231 cells. * indicates statistical significance, *p* < 0.05, *p* < 0.01 (**), *p* < 0.001 (***).

Cyclin B1 binds to and activates CDK1, forming the Cyclin B1-CDK1 complex, which is essential for driving the cell cycle transition from G2 to M phase. This complex regulates key mitotic events, such as nuclear envelope breakdown and spindle assembly, ensuring proper cell division [15]. Since **7i** inhibits MDA-MB-231 cell proliferation and is associated with G2/M phase arrest during mitosis, we further tested Cyclin B1 and CDK1 expression levels by Western blot. As shown in Figure 6A–C, after treating with **7i** for 36 h, Western blot analysis revealed that **7i** downregulated the expression of Cyclin B1 and CDK1 in MDA-MB-231 cells in a concentration-dependent manner. However, **7i** failed to attenuate the expression of these proteins in RXRα-knockout MDA-MB-231 cells. Thus, compound **7i** suppressed the expression of cyclin B1/CDK1 in an RXRα-dependent manner, thus arresting MDA-MB-231 cells in the G2/M phase. It is reported that downregulating the expression of cyclin B1 or CDK1 proteins will inhibit the mitosis of cancer cells and lead to apoptosis [16]. It is evident that compound **7i** inhibits the growth of MDA-MB-231 cells by downregulating the expression of CDK1 and Cyclin B1 and arresting the cell cycle in the G2/M phase.

### 2.5. CETSA Confirms the Binding of Compound ***7i*** to RXRα

**Figure 7:**
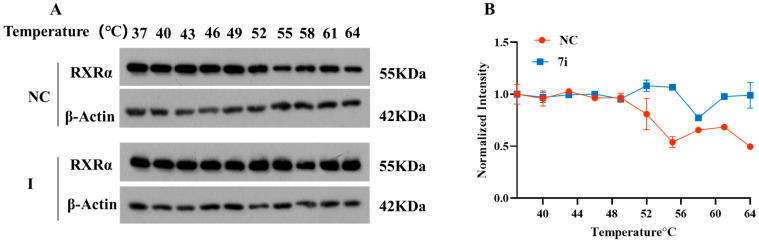
CETSA confirming the interaction between **7i** and RXRα protein. (**A**) MDA-MB-231 cells were treated with **7i** (50 μM) or DMSO control (NC) for 2 h, heated to the indicated temperatures, and lysed. RXRα was detected by Western blot analysis. (**B**) Analysis of RXRα expression levels by grayscale measurement with GraphPad Prism (Version 10.2.4).

The Cellular Thermal Shift Assay (CETSA) is a technique used to study the interaction between drugs (or small molecules) and target proteins in living cells, which verifies binding by detecting temperature-induced changes in protein thermal stability [17]. The CETSA experiment was further employed to verify the binding ability between **7i** and RXRα. As depicted in Figure 7A,B, the MDA-MB-231 cells were incubated with DMSO (NC group) or **7i** (treatment group) for 2 h. The CETSA profile revealed a significant thermal stabilization of RXRα upon treatment with **7i**, suggesting the direct binding of **7i** to RXRα.

### 2.6. Molecular Docking

**Figure 8:**
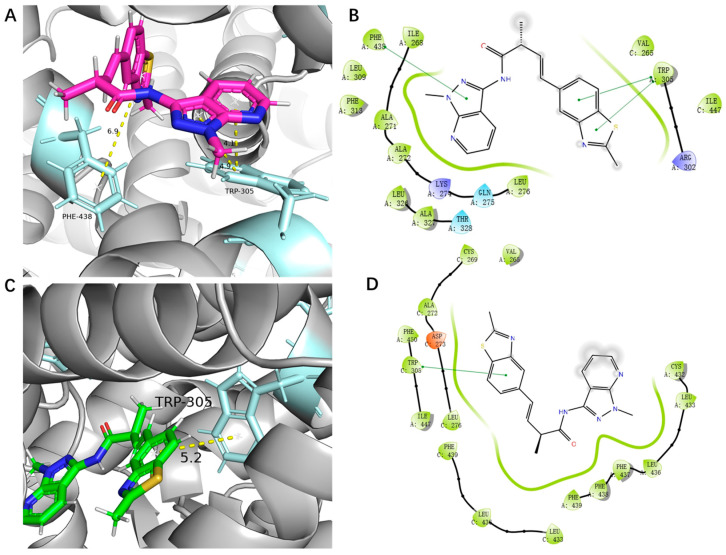
The molecule docking of **7i** (*R*-**7i** and *S*-**7i**) with RXRα-LBD (PDB code: 4N8R) [20]. (**A**) The 3D interaction diagram of *R*-**7i** with RXRα. The yellow dotted line represents pi–pi interactions; *R*-**7i** is shown as a magenta line, and amino acids are shown as pale cyan lines. (**B**) The 2D diagram of noncovalent interactions between *R*-**7i** and RXRα. (**C**) The 3D interaction diagram of *S*-**7i** with RXRα. The yellow dotted line represents pi–pi interactions, *S***-7i** is shown as a green line, and amino acids are shown as pale cyan lines. (**D**) The 2D diagram of noncovalent interactions between *S*-**7i** and RXRα.

Molecular docking is a computational simulation technique used to predict interactions between molecules (e.g., proteins and ligands) and identify potential target proteins of compounds, which is widely applied in drug discovery and development [18,19]. The above results demonstrated that **7i** inhibited the proliferation of MDA-MB-231 cells in an RXRα-dependent manner. Therefore, **7i** exerts its anti-tumor activity by binding to RXRα. Compound **7i** is a pair of racemic mixtures (*R*-**7i** and *S*-**7i**) with one chiral center. We performed molecular docking studies to evaluate the binding abilities of *R*-**7i** and *S*-**7i** to RXRα, respectively. As shown in Figure 8, *R*-**7i** and *S-***7i** could bind to RXRα by pi–pi interaction between Trp305 and benzothiazole nucleus, which is similar to meroterpenthiazole A (Figure 8A–D). Moreover, the introduction of a pyrazolo[3,4-b] pyridine moiety into the benzothiazole side chain provided an additional pi–pi interaction between *R*-**7i** and RXRα, which enhanced the binding affinity (Figure 8A,B). Both R-7i and S-7i exhibit hydrophobic interactions (Figure 8A–D).

Furthermore, the binding free energy of *R*-**7i** and *S-***7i** towards RXRα was −41.84 kcal/mol and −51.49 kcal/mol, respectively, while the binding energy of classical ligand K8008 was −54.66 kcal/mol. Therefore, both the *R*-**7i** and *S*-**7i** isomers showed high binding affinity to RXRα. Compound **7b** did not inhibit RXRα transcriptional activity and showed no cytotoxicity against MDA-MB-231 cells. Unlike **7i**, **7b** (*R*-**7b** and *S*-**7b**) did not exhibit pi–pi stacking or hydrogen bonding interactions with RXRα. This difference in behavior may be attributed to structural distinctions between the two compounds. Compound **7b** contains a saturated five-membered ring substituent on the amide side chain, while **7i** features an unsaturated conjugated pyridopyrazole ring. The extended conjugated system in **7i** facilitates enhanced pi–pi stacking interactions with RXRα, likely contributing to its anti-cancer activities (Figure S64).

## 3. Materials and Methods

### 3.1. Instruments

All chemicals were purchased commercially. The progress of the organic chemical reaction was monitored by thin-layer chromatography (TLC) (Qingdao Haiyang Chemical, Qingdao, China). ^1^H NMR (600 MHz) and ^13^C NMR (150 MHz) spectra were obtained using the Bruker AV2 600 Ultra shield spectrometer; chemical shifts (δ) are noted in ppm using tetramethyl silane (TMS) as an internal standard. The high-resolution mass spectra (HRMS) were recorded with the Thermo QExactive Mass spectrometer (Waltham, MA, USA) equipped with an electrospray ionization source (ESI). The products were purified using silica gel (Qingdao Haiyang Chemical, Qingdao, China, 200–300 mesh) as the stationary phase for separation. The purity of all compounds was detected an Agilent 1260 system (Santa Clara, CA, USA), and a Shimadzu LC-16P system was used for HPLC analysis (Kyoto, Japan).

### 3.2. Chemical Synthesis

#### 3.2.1. General Procedure for Carboxylic Acid Benzothiazole Derivatives (**3a**–**3b** and **6a**–**6b**)

Bromobenzothiazole (1 g, 4.6800 mmol), tri(o-tolyl) phosphine (427.20 mg, 1.4100 mmol), and palladium acetate (51.70 mg, 0.4850 mmol) were weighed and placed in a 100 mL round-bottom flask. The reaction system was rendered anhydrous and oxygen-free. Under continuous stirring, 20.0 mL of the reaction solvent (a 3:1 mixture of 1,4-dioxane and water) was added slowly, followed by the addition of triethylamine (1.3 mL, 9.3600 mmol). The reaction was then allowed to proceed for 24 h.

Upon completion of the reaction, the mixture was washed three times with 0.1 mol/L sodium hydroxide solution. The aqueous phase was then extracted three times with ethyl acetate. The combined organic layers were washed twice with saturated brine and dried over anhydrous sodium sulfate. After concentration under reduced pressure, the crude product was obtained. Purification by flash column chromatography (petroleum ether/dichloromethane = 10:1) afforded the final products **3a**–**3b** and **6a**–**6b** [21].

##### (E)-4-(benzo[d]thiazol-6-yl)-2-methylbut-3-enoic Acid (**3a**)

Compound **3a** was synthesized according to the general procedure A and was a white solid, 38.5 mg, yield: 75%. HR-ESI-MS: m/z [M+H]^+^ calcd for 234.0583 (C_12_H_11_NO_2_S, found, 234.0584). ^1^HNMR (600 MHz, CDCl_3_) *δ* 9.00 (s, 1 H, H-2), 8.07 (d, *J* = 8.4 Hz, 1 H, H-5), 7.92 (d, *J* = 1.2 Hz,1 H, H-8), 7.58 (dd, *J* = 1.2, 8.4 Hz, 1 H, H-6), 6.65 (d, *J* = 16.2 Hz, 1 H, H-10), 6.42 (dd, *J* = 16.2, 7.8 Hz, 1 H, H-11), 3.40 (m,1 H, H-12), 1.44 (d, *J* = 6.6 Hz, 3 H, H-14). ^13^C NMR (151 MHz, CDCl_3_) *δ* 179.9 (C-13), 154.6 (C-2), 152.45 (C-4), 134.9 (C-9), 134.4 (C-8), 130.9 (C-5), 129.6 (C-7), 124.9 (C-6), 123.5 (C-11), 119.8 (C-10), 43.2 (C-12), 17.4 (C-14).

##### (E)-2-methyl-4-(2-methylbenzo[d]thiazol-6-yl)-3-enoic Acid (**3b**)

Compound **3b** was synthesized according to the general procedure A and was a white solid, 29.8 mg, yield: 70%. HR-ESI-MS: m/z [M+H]^+^ calcd for 248.0740 (C_13_H_13_NO_2_S, found, 248.0737). ^1^H NMR (600 MHz, CD_3_OD) *δ* 7.84 (d *J* = 1.2 Hz, 1H, H-8), 7.75 (d, *J* = 8.4 Hz, 1 H, H-5), 7.50 (dd, *J* = 1.2, 8.4 Hz, 1 H, H-6), 6.55 (d, *J* = 16.2 Hz, 1 H, H-10), 6.36 (dd, *J* = 7.8, 16.2 Hz, 1 H, H-11), 3.27 (m,1 H, H-12), 2.75 (s, 3H, H-15), 1.33 (d, *J* = 6.6 Hz, 3 H, H-14). ^13^C NMR (151 MHz, CD_3_OD) *δ* 178.2 (C-13), 169.9 (C-2), 153.3 (C-4), 137.2 (C-9), 135.9 (C-8), 131.4 (C-5), 131.2(C-7), 125.6 (C-6), 122.6 (C-11), 120.4 (C-10), 44.4 (C-12), 19.7 (C-15), 17.9 (C-14).

##### (E)-4-(benzo[d]thiazol-5-yl)-2-methylbut-3-enoic Acid (**6a**)

Compound **6a** was synthesized according to the general procedure A and was a white solid, 27.7 mg, yield: 73%. HR-ESI-MS: m/z [M-H]^−^ calcd for 232.0438 (C_12_H_11_NO_2_S, found, 232.0434). ^1^H NMR (600 MHz, CDCl_3_) *δ* 9.03 (s,1H, H-2), 8.11 (d *J* = 1.2 Hz,1H, H-5), 7.88 (d, *J* = 8.4 Hz, 1H, H-8), 7.53 (dd, *J* = 1.2, 8.4 Hz, 1H, H-7), 6.68 (d, 16.2 Hz, 1H, H-10), 6.43 (dd, *J* = 7.8, 16.2 Hz, 1H, H-11), 3.42 (m,1H, H-12), 1.45 (d, *J* = 6.6 Hz, 3H, H-14). ^13^C NMR (151 MHz, CDCl_3_) *δ* 179.6 (C-13), 155.1 (C-2), 153.4 (C-4), 135.8 (C-9), 132.7 (C-6), 131.0 (C-7), 129.5 (C-8), 124.1 (C-5), 121.9 (C-10), 121.3 (C-11), 43.2 (C-12), 17.4 (C-14).

##### (E)-2-methyl-4-(2-methylbenzo[d]thiazol-5-yl) but-3-enoic Acid (**6b**)

Compound **6b** was synthesized according to the general procedure A and was a white solid, 21.7 mg, yield: 69%. (E)-2-methyl-4-(2-methylbenzo[d]thiazol-5-yl) but-3-enoic acid. HR-ESI-MS: m/z [M+H]^+^ calcd for 248.0740 (C_13_H_13_NO_2_S, found, 248.0739). ^1^H NMR (600 MHz, CDCl_3_) *δ* 7.94 (d, *J* = 1.2 Hz, 1H, H-5), 7.73 (d, *J* = 8.4 Hz, 1H, H-8), 7.41 (dd, *J* = 1.2, 8.4 Hz, 1H, H-7), 6.64 (d, *J* = 16.2 Hz, 1H, H-10), 6.42 (dd, *J* = 7.8, 16.2 Hz, 1H, H-11), 3.40 (m, 1H, H-12), 2.85 (s, 3H, H-15), 1.44 (d, *J* = 6.6 Hz, 3H, H-14). ^13^C NMR (151 MHz, CDCl_3_) *δ* 179.4 (C-13), 168.7 (C-2), 153.3 (C-4), 135.5 (C-9), 134.3 (C-6), 131.0 (C-7), 129.2 (C-8), 123.4 (C-5), 121.4 (C-10), 119.9 (C-11), 43.2 (C-12), 20.0 (C-15), 17.3 (14C).

#### 3.2.2. General Procedure for Amide Benzothiazole Derivatives (**4a**–**4b** and **7a**–**7i**)

The carboxylate product from the previous step (0.0857 mmol), the amine reactant (0.1028 mmol), N-methylimidazole (24.62 mg, 0.3000 mmol), and N, N, N, N-tetramethylchloroformamidinium hexafluorophosphate (26.45 mg, 0.0930 mmol) were weighed and placed in a 10 mL round-bottom flask. Acetonitrile (1 mL) was added, and the reaction mixture was stirred under monitoring by TLC.

Upon completion of the reaction, the mixture was washed with 0.1 mol/L hydrochloric acid to remove basic and water-soluble impurities. The aqueous layer was extracted three times with ethyl acetate, and the combined organic layers were subsequently washed three times with saturated brine. The organic phase was dried over anhydrous sodium sulfate, concentrated under reduced pressure to afford the crude product, and finally purified by silica gel column chromatography to yield the target compounds **4a**–**4h** and **7a**–**7i** [22]. Following the aforementioned reactions, 21 novel benzothiazole derivatives were successfully synthesized. The structures of all compounds were confirmed by ^1^H NMR, ^13^C NMR, and HR-ESI-MS analyses, as detailed below.

##### (E)-4-(benzo[d]thiazol-6-yl)-N-cyclopropyl-2-methylbut-3-enamide (**4a**)

Compound **4a** was synthesized according to the general procedure B and was a white solid, 29.8 mg, yield: 70%. HR-ESI-MS: m/z [M+H]^+^ calcd for 273.1056 (C_15_H_16_N_2_OS, found, 273.1054). ^1^H NMR (600 MHz, CD_3_OD) *δ* 9.17 (s, 1H, H-2), 8.04 (d, *J* = 8.4 Hz, 1H, H-8), 8.01 (s, 1H, H-5), 7.61 (d, *J* = 8.4 Hz, 1H, H-6), 6.63 (dd, *J* = 15.6 Hz, 1H, H-10), 6.4 (dd, *J* = 8.4, 15.6 Hz, 1H, H-11), 3.20 (m, 1H, H-14), 2.68 (m, 1H, H-12), 1.31 (d, *J* = 6.6 Hz, 3H, H-17), 0.72 (m, 2H, H-15), 0.50 (m, 1H, H-16). ^13^C NMR (151 MHz, CD_3_OD) *δ* 177.3 (C-13), 155.6 (C-2), 152.0 (C-4), 135.3 (C-9), 134.2 (C-7), 131.0 (C-6), 129.8 (C-8), 124.4 (C-5), 122.3 (C-10), 119.5 (C-11), 44.2 (C-14), 22.1 (C-18), 16.9 (C-12), 5.1 (C-17), 5.0 (C-15, 16).

##### (E)-4-(benzo[d]thiazol-6-yl)-N-cyclopentyl-2-methylbut-3-enamide (**4b**)

Compound **4b** was synthesized according to the general procedure B and was a white solid, 20.8 mg, yield: 66%. HR-ESI-MS: m/z [M+H]^+^ calcd for 301.1369 (C_17_H_20_N_2_OS, found, 301.1367). ^1^H NMR (600 MHz, CD_3_OD) *δ* 9.16 (s, 1H, H-2), 8.02 (s, 1H, H-8), 7.97 (d, *J* = 8.4 Hz, 1H, H-5), 7.6 (d, *J* = 8.4 Hz, 1H, H-6), 6.64 (d, *J* = 15.6 Hz, 1H, H-10), 6.44 (dd, *J* = 8.4, 15.6 Hz, 1H, H-11), 4.13 (m, 1H, H-14), 3.25 (m, 1H, H-12), 1.96 (m, 2H, H-15), 1.74 (m, 2H, H-16), 1.6 (m, 2H, H-17), 1.49 (m, 2H, H-18), 1.32 (d, *J* = 7.2 Hz, 3H, H19). ^13^C NMR (151 MHz, CD_3_OD) *δ* 175.3 (C-13), 155.5 (C-2), 152.0 (C-4), 135.3 (C-9), 134.2 (C-7), 131.4 (C-6), 129.6 (C-8), 124.4 (C-5), 122.4 (C-10), 119.4 (C-11), 51.0 (14C), 44.3 (C-12), 32.1 (C-15, 18), 23.4 (C-16, 17), 17.0 (C-19).

##### (E)-N-cyclopentyl-2-methyl-4- (2-methylbenzo[d]thiazol-6-yl) but-3-enamide (**4c**)

Compound **4c** was synthesized according to the general procedure B and was a white solid, 30.6 mg, yield: 71%. HR-ESI-MS: m/z [M+H]^+^ calcd for 315.1526 (C_18_H_22_N_2_OS, found, 315.1522). ^1^H NMR (600 MHz, CD_3_OD) *δ* 7.85 (s, 1H, H-8), 7.77 (d, *J* = 8.4 Hz, 1H, H-6), 7.52 (d, *J* = 8.4 Hz, 1H, H-5), 6.59 (d, *J* = 15.6 Hz, 1H, H-10), 6.39 (dd, *J* = 8.4, 15.6 Hz, 1H, H-11), 4.1 (m, 1H, H-14), 3.23 (m, 1H, H-12), 2.78 (s, 3H, H-20), 1.93 (m, 2H, H-15), 1.67 (m, 2H, H-16), 1.55 (m, 2H, H-17), 1.41 (m, 2H, H-18). ^13^C NMR (151 MHz, CD_3_OD) *δ* 176.7 (C-13), 169.8 (C-2), 153.3 (C-4), 137.2 (C-9), 136.0 (C-7), 132.3 (C-6), 131.1 (C-8), 125.6 (C-5), 122.7 (C-10), 120.3 (C-11), 52.4 (C-14), 45.7 (C-12), 33.5 (C-20), 24.8 (C-15, 18), 19.7 (C-16, 17), 18.5 (C-19).

##### (E)-4-(benzo[d]thiazol-6-yl)-N-cyclohexyl-2-methylbut-3-enamide (**4d**)

Compound **4d** was synthesized according to the general procedure B and was a white solid, 40.7 mg, yield: 78%. HR-ESI-MS: m/z [M+H]^+^ calcd for 315.1526 (C_18_H_22_N_2_OS, found, 315.1521). ^1^H NMR (600 MHz, CDCl_3_) *δ* 8.95 (s, 1H, H-2), 8.06 (d, *J* = 8.4 Hz, 1H, H-5), 7.91 (d, *J* = 1.2 Hz, 1H, H-8), 7.56 (d, *J* = 1.2, 8.4 Hz, 1H, H-6), 6.62 (d, *J* = 15.6 Hz, 1H, H-10), 6.38 (dd, *J* = 8.4, 15.6 Hz, 1H, H-11), 5.49 (br, 1H, -NH-), 3.80 (m, 1H, H-14), 3.14 (m, 1H, H-12), 1.95 (m, 2H, H-15), 1.71 (m, 2H, H-16), 1.62 (m, 2H, H-17), 1.38 (d, *J* = 7.2 Hz, 3H, H-20), 1.3 (m, 2H, H-18), 1.16 (m, 2H, H-19). ^13^C NMR (151 MHz, CDCl_3_) *δ* 172.7 (C-13), 154.1 (C-2), 152.7 (C-4), 134.7 (C-9), 134.4 (C-7), 131.2 (C-6), 130.8 (C-8), 124.7 (C-5), 123.5 (C-10), 119.6 (C-11), 48.3 (C-14), 45.2 (C-12), 33.2 (C-15, 18), 25.6 (C-16, 19), 24.9 (C-17), 17.7 (C-20).

##### (E)-4-(benzo[d]thiazol-6-yl)-N-(2,2-dimethoxyethyl)-2-methylbut-3-enamide (**4e**)

Compound **4e** was synthesized according to the general procedure B and was a white solid, 23.7 mg, yield: 70%. HR-ESI-MS: m/z [M-H]^−^ calcd for 319.1122 (C_16_H_20_N_2_O_3_S, found, 319.1123). ^1^H NMR (600 MHz, CDCl_3_) *δ* 8.95 (s, 1H, H-2), 8.06 (d, *J* = 8.4 Hz, 1H, H-5), 7.91 (d, *J* = 1.2, 1H, H-8), 7.56 (dd, *J* = 1.2, 8.4 Hz, 1H, H-6), 6.64 (d, *J* = 16.2 Hz, 1H, H-10), 6.36 (dd, *J* = 7.8, 16.2 Hz, 1H, H-11), 5.85 (br, 1H, -NH-), 4.38 (t, 1H, H-15), 3.43 (m, 2H, H-14), 3.38 (s, 3H, H-16), 3.37 (s, 3H, H-17), 3.18 (m, 1H, H-12), 1.39 (d, *J* = 6.6 Hz, 3H, H-18). ^13^C NMR (151 MHz, CDCl_3_) *δ* 174.1 (C-13), 154.2 (C-2), 152.9 (C-4), 134.7 (C-9), 134.5 (C-7), 131.1 (C-6), 130.9 (C-8), 124.7 (C-5), 123.6 (C-10), 119.7 (C-11), 102.8 (C-15), 54.7 (C-16), 54.7 (C-17), 45.1 (C-14), 41.2 (C-12), 17.7 (C-18).

##### (E)-N-(2,2-dimethoxyethyl)-2-methyl-4-(2-methylbenzo[d]thiazol-6-yl) but-3-enamide (**4f**)

Compound **4f** was synthesized according to the general procedure B and was a white solid, 26.7 mg, yield: 74%. HR-ESI-MS: m/z [M-H]^−^ calcd for 333.1278 (C_17_H_22_N_2_O_3_S, found, 333.1281). ^1^H NMR (600 MHz, CDCl_3_) *δ* 7.86 (d, *J* = 8.4 Hz, 1H, H-5), 7.78 (d, *J* = 1.2, 1H, H-8), 7.46 (dd, *J* = 1.2, 8.4 Hz, 1H, H-6), 6.59 (d, 16.2 Hz, 1H, H-10), 6.30 (dd, *J* = 7.8, 16.2 Hz, 1H, H-11), 5.87 (br, 1H, -NH-), 4.38 (t, 1H, H-15), 3.39 (m, 2H, H-14), 3.37 (s, 3H, H-16), 3.36 (s, 3H, H-17), 3.16 (m, 1H, H-12), 2.81 (s, 3H, H-19), 1.38 (d, *J* = 6.6 Hz, 3H, H-18). ^13^C NMR (151 MHz, CDCl_3_) *δ* 174.1 (C-13), 167.4 (C-2), 153.0 (C-4), 136.4 (C-9), 133.8 (C-8), 131.3 (C-6), 130.2 (C-7), 124.5 (C-5), 122.4 (C-10), 119.2 (C-11), 102.8 (C-15), 54.7 (C-16), 54.6 (C-17), 45.1 (C-14), 41.2 (C-12), 20.3 (C-19), 17.7 (C-18).

##### (E)-4-(benzo[d]thiazol-6-yl)-2-methyl-N-(1-methyl-1H-pyrazolo[3,4-b] pyridin-3-yl) but-3-enamide (**4g**)

Compound **4g** was synthesized according to the general procedure B and was a white solid, 28.7 mg, yield: 76%. HR-ESI-MS: m/z [M-H]^−^ calcd for 362.1081 (C_19_H_17_N_5_OS, found, 362.1083). ^1^H NMR (600 MHz, CDCl_3_) *δ* 8.97 (s, 1H, H-2), 8.61 (dd, *J* = 1.2, 8.4 Hz, 1H, H-6), 8.51 (dd, *J* = 1.2, 8.4 Hz, 1H, H-21), 8.06 (d, *J* = 8.4 Hz, 1H, H-20), 7.92 (d, *J* = 1.2, 1H, H-8), 7.58 (dd, *J* = 1.2, 8.4 Hz, 1H, H-19), 7.08 (d, *J* = 8.4 Hz, 1H, H-5), 6.75 (d, *J* = 16.2 Hz, 1H, H-10), 6.45 (dd, *J* = 7.8, 16.2 Hz, 1H, H-11), 4.01 (s, 3H, H-23), 3.47 (m, 1H, H-12), 1.53 (d, *J* = 6.6 Hz, 3H, H-24). ^13^C NMR (151 MHz, CDCl_3_) *δ* 171.7 (C-13), 154.4 (C-17), 153.0 (C-2), 150.7 (C-14), 149.7 (C-19), 138.2 (C-4), 134.5 (C-9), 134.3 (C-22), 134.2 (C-21), 132.4 (C-20), 129.7 (C-7), 124.8 (C-8), 123.7 (C-6), 119.9 (C-5), 116.4 (C-10), 108.4 (C-11), 45.3 (C-23), 33.7 (C-12), 17.5 (C-24).

##### (E)-4-(benzo[d]thiazol-6-yl)-2-methyl-N-(1-methyl-1H-pyrazolo[3,4-b] pyridin-3-yl) but-3-enamide (**4h**)

Compound **4h** was synthesized according to the general procedure B and was a white solid, 32.7 mg, yield: 75%. HR-ESI-MS: m/z [M-H]^−^ calcd for 376.1238 (C_20_H_19_N_5_OS, found, 376.1237). ^1^H NMR (600 MHz, CDCl_3_) *δ* 8.60 (dd, *J* = 1.2, 8.4 Hz, 1H, H-19), 8.49 (dd, *J* = 1.2, 8.4 Hz, 1H, H-6), 8.40 (br, 1H, -NH-), 7.86 (d, *J* = 8.4 Hz, 1H, H-20), 7.79 (d, *J* = 1.2 Hz, 1H, H-21), 7.48 (d, *J* = 1.2 Hz, 1H, H-8), 7.08 (dd, *J* = 8.4 Hz 1H, H-5), 6.70 (d, *J* = 16.2 Hz, 1H, H-10), 6.38 (dd, *J* = 7.8, 16.2 Hz, 1H, H-11), 4.00 (s, 3H, H-23), 3.44 (m, 1H, H-12), 2.81 (s, 3H, H-25), 1.51 (d, *J* = 6.6 Hz, 3H, H-24). ^13^C NMR (151 MHz, CDCl_3_) *δ* 171.8 (C-13), 167.6 (C-17), 153.1 (C-2), 150.7 (C-19), 149.7 (C-14), 138.2 (C-4), 136.4 (C-9), 134.2 (C-22), 133.3 (C-7), 132.5 (C-8), 129.1 (C-6), 124.6 (C-5), 122.4 (C-20), 119.4 (C-21), 116.3 (C-10), 108.4 (C-11), 45.3 (C-23), 33.6 (C-12), 20.3 (C-24), 17.5 (C-25).

##### (E)-N-cyclopropyl-2-methyl-4- (2-methylbenzo[d]thiazol-5-yl) but-3-enamide (**7a**)

Compound **7a** was synthesized according to the general procedure B and was a white solid, 21.2 mg, yield: 68%. HR-ESI-MS: m/z [M+H]^+^ calcd for 287.1213 (C_16_H_18_N_2_OS, found, 287.1210). ^1^H NMR (600 MHz, CD_3_OD) *δ* 7.82 (d, *J* = 8.4 Hz, 1H, H-8), 7.81 (s, 1H, H-5), 7.45 (d, *J* = 8.4 Hz, 1H, H-7), 6.61 (d, *J* = 15.6 Hz, 1H, H-10), 6.40 (dd, *J* = 8.4, 15.6 Hz, 1H, H-11), 3.20 (m, 1H, H-14), 2.70 (m, 1H, H-12), 1.3 (d, *J* = 6.6 Hz, 3H, H-17), 0.74 (m, 2H, H-15), 0.52 (m, 2H, H-16). ^13^C NMR (151 MHz, CD_3_OD) *δ* 178.7 (C-13), 170.4 (C-2), 154.5 (C-4), 137.2 (C-9), 135.5 (C-8), 131.9 (C-7), 131.5 (C-6), 124.3 (C-5), 122.7 (C-10), 120.4 (C-11), 45.6 (C-14), 23.5 (C-18), 19.7 (C-15), 18.3 (C-16), 6.5 (C-17).

##### (E)-4-(benzo[d]thiazol-5-yl)-N-cyclopentyl-2-methylbut-3-enamide (**7b**)

Compound **7b** was synthesized according to the general procedure B and was a white solid, 26.5 mg, yield: 73%. HR-ESI-MS: m/z [M+H]^+^ calcd for 301.1369 (C_17_H_20_N_2_OS, found, 301.1366). ^1^H NMR (600 MHz, CD_3_OD) *δ* 9.21 (s, 1H, H-2), 8.01 (d, *J* = 8.4 Hz, 1H, H-8), 7.99 (s, 1H, H-5), 7.58 (d, *J* = 8.4 Hz, 1H, H-7), 7.56, 6.66 (d, *J* = 15.6 Hz, 1H, H-10), 6.46 (dd, *J* = 8.4, 15.6 Hz, 1H, H-11), 4.14 (m, 1H, H-14), 3.26 (m, 1H, H-12), 1.93 (m, 2H, H-15), 1.72 (m, 1H, H-16), 1.59 (m, 1H, H-17), 1.47 (m, 1H, H-18), 1.33 (d, *J* = 7.8 Hz, 3H, H-19). ^13^C NMR (151 MHz, CD_3_OD) *δ* 175.4 (C-13), 156.1 (C-2), 153.3 (C-4), 136.1 (C-9), 132.4 (C-6), 131.1 (C-7), 129.8 (C-8), 123.6 (C-5), 121.8 (C-10), 120.1 (C-11), 51.0 (C-14), 44.3 (C-15, 18), 32.1 (C-17), 23.4 (C-16), 17.0 (C-19).

##### (E)-N-cyclopentyl-2-methyl-4- (2-methylbenzo[d]thiazol-5-yl) but-3-enamide (**7c**)

Compound **7c** was synthesized according to the general procedure B and was a white solid, 35.5 mg, yield: 75%. HR-ESI-MS: m/z [M+H]^+^ calcd for 315.1523 (C_18_H_22_N_2_OS, found, 315.1522). ^1^H NMR (600 MHz, CD_3_OD) *δ* 7.81 (d, *J* = 8.4 Hz, 1H, H-8), 7.79 (s, 1H, H-5), 7.46 (d, *J* = 8.4 Hz, 1H, H-7), 6.61 (d, *J* = 15.6 Hz, 1H, H-10), 6.41 (dd, *J* = 8.4, 15.6 Hz, 1H, H-11), 4.13 (m, 1H, H-14), 3.24 (m, 1H, H-12), 3.19 (s, 3H, H-20), 1.96 (m, 2H, H-15), 1.75 (m, 2H, H-16), 1.62 (m, 2H, H-17), 1.49 (m, 2H, H-18), 1.31 (d, *J* = 6.6 Hz, 3H, H-19). ^13^C NMR (151 MHz, CD3OD) *δ* 176.7 (C-13), 170.4 (C-2), 154.5 (C-4), 137.3 (C-9), 135.5 (C-6), 132.2 (C-7), 131.3 (C-8), 124.3 (C-5), 122.7 (C-10), 120.4 (C-11), 52.4 (C-14), 45.6 (C-20), 33.5 (C-12), 24.8 (C-15, 18), 19.7 (C-16, 17), 18.4 (C-19).

##### (E)-4-(benzo[d]thiazol-5-yl)-N-cyclohexyl-2-methylbut-3-enamide (**7d**)

Compound **7d** was synthesized according to the general procedure B and was a white solid, 37.5 mg, yield: 76%. HR-ESI-MS: m/z [M+H]^+^ calcd for 315.1526 (C_18_H_22_N_2_OS, found, 315.1524). ^1^H NMR (600 MHz, CDCl_3_) *δ* 9.00 (s, 1H, H-2), 8.08 (s, 1H, H-5), 7.89 (d, *J* = 8.4 Hz, 1H, H-7), 7.53 (d, *J* = 8.4 Hz, 1H, H-8), 6.66 (d, *J* = 15.6 Hz, 1H, H-10), 6.40 (dd, *J* = 8.4, 15.6 Hz, 1H, H-11), 5.51 (br, 1H, -NH-), 3.78 (m, 1H, H-14), 3.16 (m, 1H, H-12), 1.92 (m, 2H, H-15), 1.69 (m, 2H, H-16), 1.61 (m, 1H, H-17), 1.39 (d, *J* = 7.2 Hz, 2H, H-18), 1.36 (m, 2H, H-19), 1.16 (m, 3H, H-20). ^13^C NMR (151 MHz, CDCl_3_) *δ* 172.8 (C-13), 154.7 (C-2), 153.8 (C-4)2, 135.7 (C-9), 132.8 (C-7), 131.1 (C-7), 131.0 (C-8), 123.9 (C-5), 122.0 (C-10), 121.5 (C-11), 48.4 (C-14), 45.2 (C-12), 33.3 (C-15, 19), 25.7 (C-16, 18), 25.0 (C-17), 17.7 (C-20).

##### (E)-N-cyclohexyl-2-methyl-4- (2-methylbenzo[d]thiazol-5-yl) but-3-enamide (**7e**)

Compound **7e** was synthesized according to the general procedure B and was a white solid, 33.5 mg, yield: 74%. HR-ESI-MS: m/z [M+H]^+^ calcd for 329.1682 (C_19_H_24_N_2_OS, found, 329.1679). ^1^H NMR (600 MHz, CDCl_3_) *δ* 7.88 (d, *J* = 8.4 Hz, 1H, H-8), 7.79 (d, *J* = 1.2 Hz, 1H, H-5), 7.48 (dd, *J* = 1.2, 8.4 Hz, 1 H, H-7), 6.58 (d, *J* = 15.6 Hz, 1H, H-10), 6.33 (dd, *J* = 8.4, 15.6 Hz, 1H, H-11), 3.77 (m, 1H, H-14), 3.13 (m, 1H, H-12), 2.83 (s, 3H, H-21), 1.92 (m, 2H, H-15), 1.69 (m, 2H, H-16), 1.61 (m, 1H, H-17), 1.37 (d, *J* = 7.2 Hz, 3H, H-20), 1.34 (m, 2H, H-19), 1.16 (m,2H, H-18). ^13^C NMR (151 MHz, CDCl_3_) *δ* 172.8 (C-13), 167.4 (C-2), 152.6 (C-4), 136.2 (C-9), 133.9 (C-6), 131.0 (C-7), 130.6 (C-8), 124.5 (C-5), 122.3 (C-10), 119.1 (C-11), 48.3 (C-14), 45.2 (C-21), 33.2 (C-12), 25.5 (C-15, 19), 24.9 (C-16, 18), 20.2 (C-17), 17.7 (C-20).

##### (E)-4-(benzo[d]thiazol-5-yl)-N-(2,2-dimethoxyethyl)-2-methylbut-3-enamide (**7f**)

Compound **7f** was synthesized according to the general procedure B and was a white solid, 31.5 mg, yield: 72%. HR-ESI-MS: m/z [M-H]^−^ calcd for 319.1122 (C_16_H_20_N_2_O_3_S, found, 319.1122). ^1^H NMR (600 MHz, CDCl_3_) *δ* 8.98(s, 1H, H-2), 8.06 (d, *J* = 1.2, 1H, H-5), 7.88 (d, *J* = 8.4 Hz, 1H, H-8), 7.50 (dd, *J* = 1.2, 8.4 Hz, 1H, H-7), 6.67(d, *J* = 16.2 Hz, 1H, H-10), 6.36 (dd, *J* = 7.8, 16.2 Hz, 1H, H-11), 5.89 (br, 1H, -NH-), 4.38 (t, 1H, H-15), 3.42 (m, 2H, H-14), 3.37 (s, 3H, H-16), 3.36 (s, 3H, H-17), 3.19 (m, 1H, H-12), 1.40 (d, *J* = 6.6 Hz, 3H, H-18). ^13^C NMR (151 MHz, CDCl_3_) *δ* 174.1 (C-13), 154.7 (C-2), 153.9 (C-4), 135.5 (C-9), 132.9 (C-6), 131.4 (C-7), 130.6 (C-10), 123.8 (C-5), 122.0 (C-8), 121.5 (C-11), 102.8 (C-15), 54.7 (C-16), 54.6 (C-17), 45.1 (C-14), 41.2 (C-12), 17.7 (C-18).

##### (E)-N-(2,2-dimethoxyethyl)-2-methyl-4-(2-methylbenzo[d]thiazol-5-yl)-3-enamide (**7g**)

Compound **7g** was synthesized according to the general procedure B and was a white solid, 30.8 mg, yield: 71%. HR-ESI-MS: m/z [M-H]^−^ calcd for 333.1278 (C_17_H_22_N_2_O_3_S, found, 333.1281). ^1^H NMR (600 MHz, CDCl_3_) *δ* 7.86 (d, *J* = 1.2 Hz, 1H, H-5), 7.73 (d, *J* = 8.4 Hz, 1H, H-8), 7.41 (dd, *J* = 1.2, 8.4 Hz, 1H, H-7), 6.63 (dd, *J* = 16.2 Hz, 1H, H-10), 6.31 (dd, *J* = 7.8, 16.2 Hz, 1H, H-11), 5.91 (br, 1H, -NH-), 4.38 (t, 1H, H-15), 3.40 (m, 2H, H-14), 3.37 (s, 3H, H-16), 3.36 (s, 3H, H-17), 3.17 (m, 1H, H-12), 2.81 (s, 3H, H-19), 1.38 (d, *J* = 6.6 Hz, 3H, H-18). ^13^C NMR (151 MHz, CDCl_3_) *δ* 174.2 (C-13), 167.9 (C-2), 153.9 (C-4), 135.1 (C-9), 134.9 (C-6), 131.6 (C-7), 130.0 (C-8), 122.9 (C-5), 121.5 (C-10), 120.4 (C-11), 102.8 (C-15), 54.7 (C-16), 54.6 (C-17), 45.0 (C-14), 41.2 (C-12), 20.3 (C-19), 17.6 (C-18).

##### (E)-4-(benzo[d]thiazol-5-yl)-2-methyl-N-(1-methyl-1H-pyrazolo[3,4-b] pyridin-3-yl) but-3-enamide (**7h**)

Compound **7h** was synthesized according to the general procedure B and was a white solid, 47.8 mg, yield: 78%. HR-ESI-MS: m/z [M-H]^−^ calcd for 362.1081 (C_19_H_17_N_5_OS, found, 362.1084). ^1^H NMR (600 MHz, CDCl_3_) *δ* 8.99 (s, 1H, H-2), 8.60 (dd, *J* = 1.2, 8.4 Hz, 1H, H-19), 8.50 (dd, *J* = 1.2, 8.4 Hz, 1H, H-20), 8.43 (br, 1H, -NH-), 8.09 (s, 1H, H-5), 7.88 (d, *J* = 8.4 Hz, 1H, H-21), 7.52 (d, *J* = 8.4 Hz, 1H, H-8), 7.07 (dd, *J* = 1.2, 8.4 Hz, 1H, H-7), 6.78 (d, *J* = 16.2 Hz, 1H, H-10), 6.48 (dd, *J* = 7.8, 16.2 Hz, 1H, H-11), 4.00 (s, 3H, H-23), 3.47 (m, 1H, H-12), 1.53 (d, *J* = 6.6 Hz, 3H, H-24). ^13^C NMR (151 MHz, CDCl_3_) *δ* 171.8 (C-13), 154.8 (C-17), 153.9 (C-2), 150.7 (C-14), 149.7 (C-19), 138.2 (C-4), 135.1 (C-9), 134.1 (C-22), 133.2 (C-20), 132.5 (C-21), 129.5 (C-6), 123.8 (C-7), 122.0 (C-8), 121.7 (C-5), 116.3 (C-10), 108.4 (C-11), 45.2 (C-23), 33.6 (C-12), 17.5 (C-24).

##### (E)-2-methyl-N-(1-methyl-1H-pyrazolo[3,4-b] pyridin-3-yl)-4-(2-methylbenzo[d]-thiazol-5-yl) but-3-enamide (**7i**)

Compound **7i** was synthesized according to the general procedure B and was a white solid, 27.3 mg, yield: 73%. HR-ESI-MS: m/z [M+H]^+^ calcd for 378.1383 (C_20_H_19_N_5_OS, found, 378.1371). ^1^H NMR (600 MHz, CDCl_3_) *δ* 8.61 (dd, *J* = 1.2, 8.4 Hz, 1H, H-19), 8.51 (dd, *J* = 1.2, 8.4 Hz, 1H, H-21), 8.25 (br, 1H, -NH-), 7.91 (s, 1H, H-5), 7.76 (d, *J* = 8.4 Hz, 1H, H-20), 7.43 (d, *J* = 8.4 Hz, 1 H, H-8), 7.08 (dd, *J* = 1.2, 8.4 Hz, 1H, H-7), 6.77 (d, *J* = 16.2 Hz, 1H, H-10), 6.44 (dd, *J* = 7.8, 16.2 Hz, 1H, H-11), 4.01 (s, 3H, H-23), 3.45 (m, 1H, H-12) 2.83 (s, 3H, H-25), 1.53 (d, *J* = 6.6 Hz, 3H, H-24). ^13^C NMR (151 MHz, CDCl_3_) *δ* 171.7 (C-13), 168.0 (C-17), 153.9 (C-2), 150.7 (14-C), 149.6 (C-4), 138.2 (C-19), 135.2 (C-9), 134.8 (C-22), 134.3 (C-21), 132.9 (C-20), 129.0 (C-6), 123.0 (C-7), 121.6 (C-8), 120.6 (C-5), 116.3 (C-10), 108.4 (C-11), 45.3 (C-23), 33.7 (C-12), 20.3 (C-25), 17.5 (C-24).

### 3.3. Dual-Luciferase Reporter Assay

For this assay, 293T cells were transfected with the corresponding plasmids for 24 h, followed by compound treatment for 12 h. The cells were then lysed, and the relative luciferase activity was measured using a dual-luciferase reporter assay system according to the manufacturer’s instructions. Transfection efficiency was normalized to *Renilla* luciferase activity. DMEM medium containing 0.1% dimethyl sulfoxide (DMSO) served as a blank control. UVI3003 (1 µM, MCE, Shanghai, China) and 9-cis (0.1 µM, MCE, Shanghai, China) were used as positive controls.

### 3.4. MTT Assay

MDA-MB-231 cells cultured in 96-well plates were treated with compounds at varying concentrations for 24 h. The cells were then incubated with 2 mg/mL 3-(4,5-dimethylthiazol-2-yl)-2,5-diphenyltetrazolium bromide (MTT) at 37 °C for 4 h. After removing the MTT solution, 150 μL dimethyl sulfoxide (DMSO) was added to each well to rapidly dissolve the formazan crystals. Absorbance was measured at 492 nm.

### 3.5. Cell Cycle Analysis

MDA-MB-231 cells were harvested and fixed at −4 °C for at least 16 h with 70% ethanol. The fixed cells were co-incubated with RNase A and PI for 30–60 min at room temperature under dark conditions, prior to analysis via flow cytometry. Cell cycle distribution profiles were quantified using ModFit LT 5.0 software.

### 3.6. Western Blot

MDA-MB-231 cells were treated with compound **7i** at concentrations of 0, 20, and 30 μM for 36 h before collection. The harvested cells were resuspended in RIPA buffer supplemented with 1× phosphatase inhibitors 1 and 2, and 1× protease inhibitor cocktail, followed by a 5 min incubation. After centrifugation for 15 min, the supernatant was collected. The protein concentration of the cleavage fragments was determined using BCA assays. Equal amounts of total protein (20 μg per sample) were separated by SDS-PAGE and transferred onto PVDF membranes. The membranes were blocked with 5% non-fat milk in TBST for 1 h at room temperature, then incubated overnight at 4 °C with the respective primary antibodies. Following washing, the membranes were incubated with horseradish peroxidase (HRP)-conjugated secondary antibodies for 1 h at room temperature. Protein signals were detected using the ECL Plus system. The following primary antibodies were used for immunoblotting: anti-RXRα (1:3000 dilution; CST, Shanghai, China), anti-CDK1(1:3000 dilution; CST, Shanghai, China), anti-CyclinB1 (1:3000 dilution; CST, Shanghai, China), and anti-β-Actin (1:30,000 dilution; Proteintech, Wuhan, China).

### 3.7. Cellular Thermal Shift Assay

The MDA-MB-231 cells were cultured for 24h. Then the cells were exposed to DMSO (control group) or compound **7i** and incubated for 2h. The cells were collected and washed with PBS containing protease inhibitors (100× protease inhibitor) to prevent protein degradation. Cells were aliquoted into multiple PCR tubes, with each tube containing an equal number of cells. The PCR tubes were placed in a thermal cycler and heated at different temperatures (from 37 °C to 64 °C) for 3 min to denature the proteins. The heated cells were resuspended in NP40 buffer, and three freeze–thaw cycles were performed with liquid nitrogen to completely lyse the cells. The lysate was centrifuged at 4 °C and 20,000× *g* for 20 min. The supernatants were collected and transferred to new microtubes. Then SDS-PAGE electrophoresis was performed, the supernatants were transferred to a PVDF membrane, and immunoblotting was conducted with specific antibodies to detect the expression level of the target protein.

### 3.8. Statistical Analysis

Statistical significance was determined by analysis of variance (ANOVA) or Student’s t-test. A *p*-value < 0.05 was considered statistically significant (*), *p* < 0.01 as highly significant (**), *p* < 0.001 as extremely significant (***), and *p* < 0.0001 as exceptionally significant (***). ‘ns’ indicates non-significance.

### 3.9. Molecular Docking

The crystal structure of RXRα (PDB ID: 4N8R) used for molecular modeling was obtained from the Protein Data Bank (https://www.rcsb.org/structure/4N8R (accessed on 10 August 2025). Protein preparation was performed using the Protein Preparation Wizard module (Schrödinger, Version 2021-2), which included the addition of hydrogen atoms, removal of water molecules and crystalline solutes, assignment of bond orders, completion of missing side chains and loops with Prime, and generation of protonation states with Epik at pH 7.0 ± 2.0. Hydrogen bonding networks were optimized, and restrained energy minimization was conducted using the OPLS4 force field.

The ligand compound **7i** (***R***-**7i** and ***S*-7i**) was prepared with the LigPrep module (Schrödinger, LLC, New York, NY, USA, 2021) under default parameters. The coactivator binding site of RXRα and the putative binding region for ***R***-**7i** and ***S*-7i** were predicted using the SiteMap tool within the Schrödinger Suite. Molecular docking was performed using the Induced Fit Docking protocol with standard settings. The binding free energy of the resulting complex was evaluated with the Prime MM-GBSA module (v3.000) employing the VSGB solvation model and OPLS4 force field. Visualization and analysis of the molecular simulation results were carried out using Maestro (Schrödinger, LLC, New York, NY, USA, 2021). The binding modes of RXRα in complex with compounds ***R***-**7i** and ***S*-7i** were rendered using PyMOL (Version 2.3, Schrödinger, LLC, New York, NY, USA).

## 4. Conclusions

In summary, inspired by the marine-derived RXRα antagonist meroterpenthiazole A, 21 benzothiazole derivatives were designed and synthesized, and their abilities to inhibit 9-cis-RA-induced RXRα transactivation were evaluated. Furthermore, we evaluated the inhibitory effect of these compounds on the proliferation of MDA-MB-231 cells. Meanwhile, the structure–activity relationships in RXRα antagonist effects, as well as anti-proliferative activities against MDA-MB-231 cells, were discussed. Compound **7i** displayed the most significant inhibitory activity, while its inhibitory activity dramatically decreased in RXRα-knockout MDA-MB-231 cell lines, suggesting that the anti-cancer activities of **7i** against MDA-MB-231 cells acts in an RXRα-dependent manner. In addition, it was confirmed that **7i** blocked the division of MDA-MB-231 cells by cell cycle arrest at the G2/M phase. In contrast, a significant decrease in the proportion of G2/M phase cells was observed in RXRα-knocked-out MDA-MB-231 cells, suggesting that the induction of G2/M phase arrest by **7i** is dependent on RXRα. Furthermore, the G2/M cycle-related proteins Cyclin B1 and CDK1 showed concentration-dependent downregulation in wild-type MDA-MB-231 cells, while there was no difference in RXRα-knocked-out MDA-MB-231 cells. A Cellular Thermal Shift Assay (CETSA) confirmed that **7i** could bind to RXRα. Molecular docking studies further predicted the binding mode of **7i** toward RXRα. This study reveals a novel RXRα antagonist, **7i**, which targets RXRα and induces G2/M phase cell cycle arrest in MDA-MB-231 cells, showing potential as a lead candidate for the development of anti-cancer agents.

## Data Availability

Data are contained within the article and Supplementary Materials.

## Supplementary Materials

The following supporting information can be downloaded at https://www.mdpi.com/article/10.3390/md23090368/s1, Figure S1: ^1^H-NMR spectrum of compound **3a**; Figure S2: ^1^C-NMR spectrum of compound **3a**; Figure S3: HR-ESI-MS spectrum of compound **3a**; Figure S4: ^1^H-NMR spectrum of compound **3b**; Figure S5: ^1^C-NMR spectrum of compound **3b**; Figure S6: HR-ESI-MS spectrum of compound **3b**; Figure S7: ^1^H-NMR spectrum of compound **4a**; Figure S8: ^1^C-NMR spectrum of compound **4a**; Figure S9: HR-ESI-MS spectrum of compound **4a**; Figure S10: ^1^H-NMR spectrum of compound **4b**; Figure S11: ^1^C-NMR spectrum of compound **4b**; Figure S12: HR-ESI-MS spectrum of compound **4b**; Figure S13: ^1^H-NMR spectrum of compound **4c**; Figure S14: ^1^C-NMR spectrum of compound **4c**; Figure S15: HR-ESI-MS spectrum of compound **4c**; Figure S16: ^1^H-NMR spectrum of compound **4d**; Figure S17: ^1^C-NMR spectrum of compound **4d**; Figure S18: HR-ESI-MS spectrum of compound **4d**; Figure S19: ^1^H-NMR spectrum of compound **4e**; Figure S20: ^1^C-NMR spectrum of compound **4e**; Figure S21: HR-ESI-MS spectrum of compound **4e**; Figure S22: ^1^H-NMR spectrum of compound **4f**; Figure S23: ^1^C-NMR spectrum of compound **4f**; Figure S24: HR-ESI-MS spectrum of compound **4f**; Figure S25: ^1^H-NMR spectrum of compound **4g**; Figure S26: ^1^C-NMR spectrum of compound **4g**; Figure S27: HR-ESI-MS spectrum of compound **4g**; Figure S28: ^1^H-NMR spectrum of compound **4h**; Figure S29: ^1^C-NMR spectrum of compound **4h**; Figure S30: HR-ESI-MS spectrum of compound **4h**; Figure S31: ^1^H-NMR spectrum of compound **6a**; Figure S32: ^1^C-NMR spectrum of compound **6a**; Figure S33: HR-ESI-MS spectrum of compound **6a**; Figure S34: ^1^H-NMR spectrum of compound **6b**; Figure S35: ^1^C-NMR spectrum of compound **6b**; Figure S36: HR-ESI-MS spectrum of compound **6b**; Figure S37: ^1^H-NMR spectrum of compound **7a**; Figure S38: ^1^C-NMR spectrum of compound **7a**; Figure S39: HR-ESI-MS spectrum of compound **7a**; Figure S40: ^1^H-NMR spectrum of compound **7b**; Figure S41: ^1^C-NMR spectrum of compound **7b**; Figure S42: HR-ESI-MS spectrum of compound **7b**; Figure S43: ^1^H-NMR spectrum of compound **7c**; Figure S44: ^1^C-NMR spectrum of compound **7c**; Figure S45: HR-ESI-MS spectrum of compound **7c**; Figure S46: ^1^H-NMR spectrum of compound **7d**; Figure S47: ^1^C-NMR spectrum of compound **7d**; Figure S48: HR-ESI-MS spectrum of compound **7d**; Figure S49: ^1^H-NMR spectrum of compound **7e**; Figure S50: ^1^C-NMR spectrum of compound **7e**; Figure S51: HR-ESI-MS spectrum of compound **7e**; Figure S52: ^1^H-NMR spectrum of compound **7f**; Figure S53: ^1^C-NMR spectrum of compound **7f**; Figure S54: HR-ESI-MS spectrum of compound **7f**; Figure S55: ^1^H-NMR spectrum of compound **7g**; Figure S56: ^1^C-NMR spectrum of compound **7g**; Figure S57: HR-ESI-MS spectrum of compound **7g**; Figure S58: ^1^H-NMR spectrum of compound **7h**; Figure S59: ^1^C-NMR spectrum of compound **7h**; Figure S60: HR-ESI-MS spectrum of compound **7h**; Figure S61: ^1^H-NMR spectrum of compound **7i**; Figure S62: ^1^C-NMR spectrum of compound **7i**; Figure S63: HR-ESI-MS spectrum of compound **7i**; Figure S64: (**A**) 2D diagram of noncovalent interactions between *S*-**7b** and RXRα (**B**) 2D diagram of noncovalent interactions between *R*-**7b** and RXRα (**C**) 2D diagram of noncovalent interactions between *S*-**7i** and RXRα (**D**) 2D diagram of noncovalent interactions between *R*-**7i** and RXRα; Figure S65: The cytotoxicity against MDA-MB-231 and MDA-MB-231 RXRα KO cells of compound XS-060 at concentration of 5 μM. *p* < 0.01 (**), *p* < 0.001 (***).
