# Supplementary material for: Synthesis and Biological Evaluation of Marine-Inspired Benzothiazole Derivatives as Retinoid X Receptor-α Antagonists with Anti-Cancer Activities
Source: Mar Drugs. 2025 Sep 21;23(9):368. doi: 10.3390/md23090368 (PMC12471517; doi:10.3390/md23090368)
Supplement: Supplementary file 1 [file marinedrugs-23-00368-s001.zip › marinedrugs-3863276-supplementary.pdf]

Supplementary Materials

Synthesis and biological evaluation of Benzothiazole Derivatives as Novel Retinoid X Receptor-α ligands with Anti-cancer activities

Yingting Lin [1,†], Ming Peng [1,†], Renjing Yang [1,†], Guanghui Wang [1], Junjie Chen [1], Rong Ding [1], Cuiling Sun [1], Wenjing Tian [1,*] and Haifeng Chen [1,*]

[1] Fujian Provincial Key Laboratory of Innovative Drug Target, School of Pharmaceutical Sciences, Xiamen University, Xiamen 361005, China; Email: yingting301@163.com (Y.T.L.); 2080546929@qq.com (M.P.); 1605815212@qq.com (R.J.Y.); guanghui@xmu.edu.cn (G.H.W.); chenjunjie@xmu.edu.cn (J.J. C.); dingrong@xmu.edu.cn (R.D.); cuiling@xmu.edu.cn (C.L. S.)

* Correspondence: tianwj@xmu.edu.cn (W.J.T.); haifeng@xmu.edu.cn (H.F.C.)

† The authors contributed equally to this article

Content

Figure S1 ^{1}H-NMR spectrum of compound **3a**
Figure S2 ^{1}C-NMR spectrum of compound **3a**
Figure S3 HR-ESI-MS spectrum of compound **3a**
Figure S4 ^{1}H-NMR spectrum of compound **3b**
Figure S5 ^{1}C-NMR spectrum of compound **3b**
Figure S6 HR-ESI-MS spectrum of compound **3b**
Figure S7 ^{1}H-NMR spectrum of compound **4a**
Figure S8 ^{1}C-NMR spectrum of compound **4a**
Figure S9 HR-ESI-MS spectrum of compound **4a**
Figure S10 ^{1}H-NMR spectrum of compound **4b**
Figure S11 ^{1}C-NMR spectrum of compound **4b**
Figure S12 HR-ESI-MS spectrum of compound **4b**
Figure S13 ^{1}H-NMR spectrum of compound **4c**
Figure S14 ^{1}C-NMR spectrum of compound **4c**
Figure S15 HR-ESI-MS spectrum of compound **4c**
Figure S16 ^{1}H-NMR spectrum of compound **4d**
Figure S17 ^{1}C-NMR spectrum of compound **4d**
Figure S18 HR-ESI-MS spectrum of compound **4d**
Figure S19 ^{1}H-NMR spectrum of compound **4e**
Figure S20 ^{1}C-NMR spectrum of compound **4e**
Figure S21 HR-ESI-MS spectrum of compound **4e**
Figure S22 ^{1}H-NMR spectrum of compound **4f**
Figure S23 ^{1}C-NMR spectrum of compound **4f**
Figure S24 HR-ESI-MS spectrum of compound **4f**
Figure S25 ^{1}H-NMR spectrum of compound **4g**
Figure S26 ^{1}C-NMR spectrum of compound **4g**
Figure S27 HR-ESI-MS spectrum of compound **4g**
Figure S28 ^{1}H-NMR spectrum of compound **4h**
Figure S29 ^{1}C-NMR spectrum of compound **4h**
Figure S30 HR-ESI-MS spectrum of compound **4h**
Figure S31 ^{1}H-NMR spectrum of compound **6a**
Figure S32 ^{1}C-NMR spectrum of compound **6a**
Figure S33 HR-ESI-MS spectrum of compound **6a**
Figure S34 ^{1}H-NMR spectrum of compound **6b**
Figure S35 ^{1}C-NMR spectrum of compound **6b**
Figure S36 HR-ESI-MS spectrum of compound **6b**
Figure S37 ^{1}H-NMR spectrum of compound **7a**
Figure S38 ^{1}C-NMR spectrum of compound **7a**
Figure S39 HR-ESI-MS spectrum of compound **7a**
Figure S40 ^{1}H-NMR spectrum of compound **7b**
Figure S41 ^{1}C-NMR spectrum of compound **7b**

Figure S42 HR-ESI-MS spectrum of compound **7b**
Figure S43 ^{1}H-NMR spectrum of compound **7c**
Figure S44 ^{1}C-NMR spectrum of compound **7c**
Figure S45 HR-ESI-MS spectrum of compound **7c**
Figure S46 ^{1}H-NMR spectrum of compound **7d**
Figure S47 ^{1}C-NMR spectrum of compound **7d**
Figure S48 HR-ESI-MS spectrum of compound **7d**
Figure S49 ^{1}H-NMR spectrum of compound **7e**
Figure S50 ^{1}C-NMR spectrum of compound **7e**
Figure S51 HR-ESI-MS spectrum of compound **7e**
Figure S52 ^{1}H-NMR spectrum of compound **7f**
Figure S53 ^{1}C-NMR spectrum of compound **7f**
Figure S54 HR-ESI-MS spectrum of compound **7f**
Figure S55 ^{1}H-NMR spectrum of compound **7g**
Figure S56 ^{1}C-NMR spectrum of compound **7g**
Figure S57 HR-ESI-MS spectrum of compound **7g**
Figure S58 ^{1}H-NMR spectrum of compound **7h**
Figure S59 ^{1}C-NMR spectrum of compound **7h**
Figure S60 HR-ESI-MS spectrum of compound **7h**
Figure S61 ^{1}H-NMR spectrum of compound **7i**
Figure S62 ^{1}C-NMR spectrum of compound **7i**
Figure S63 HR-ESI-MS spectrum of compound **7i**
Figure S64 (A) 2D diagram of noncovalent interactions between S-**7b** and RXRα (B) 2D diagram of noncovalent interactions between R-**7b** and RXRα (C) 2D diagram of noncovalent interactions between S-**7i** and RXRα (D) 2D diagram of noncovalent interactions between R-**7i** and RXRα
Figure S65 The cytotoxicity against MDA-MB-231 and MDA-MB-231 RXRα KO cells of compound **XS-060** at concentration of 5 μM. $p<0.01$ (**), $p<0.001$ (***)

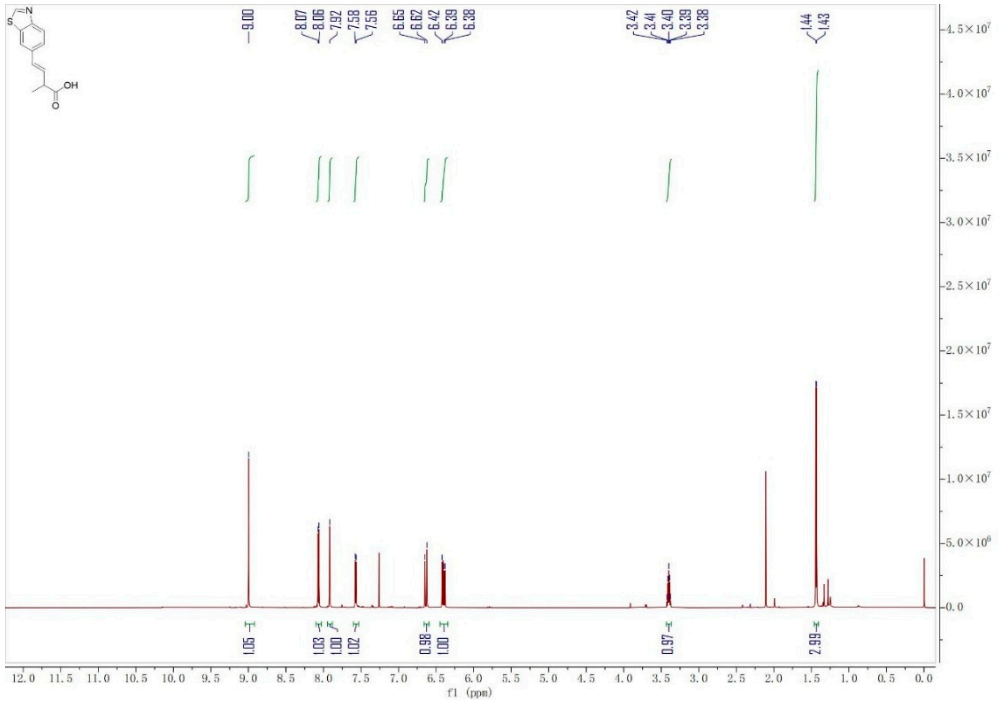

Figure S1 ^{1}H-NMR spectrum of compound **3a**

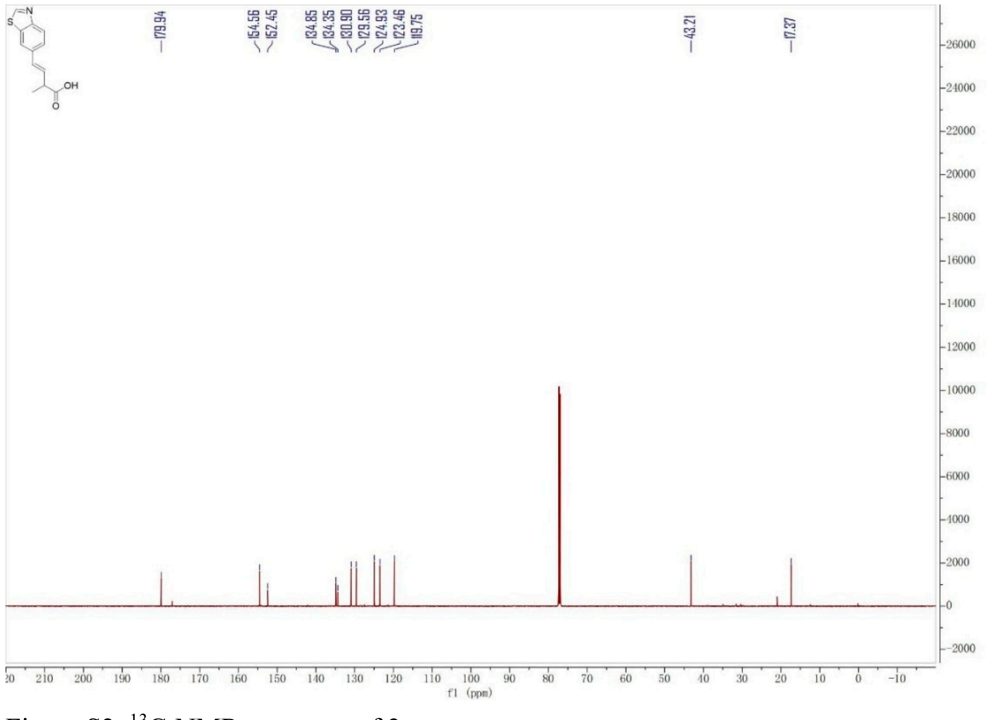

Figure S2 ^{13}C-NMR spectrum of **3a**

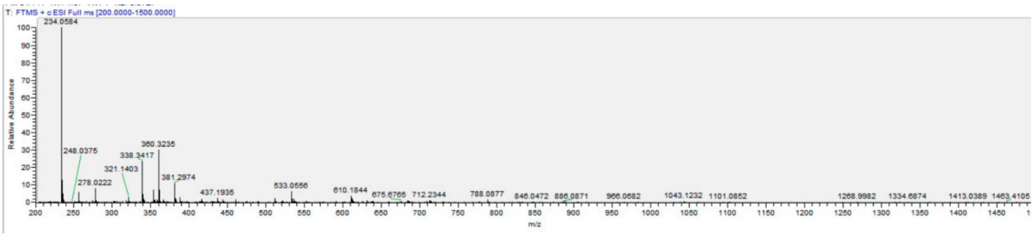

Figure S3 HR-ESI-MS spectrum of **3a**

HR-ESI-MS: m/z $[M+H]^+$ calcd for 234.0583 ($C_{12}H_{11}NO_2S$, found, 234.0584).

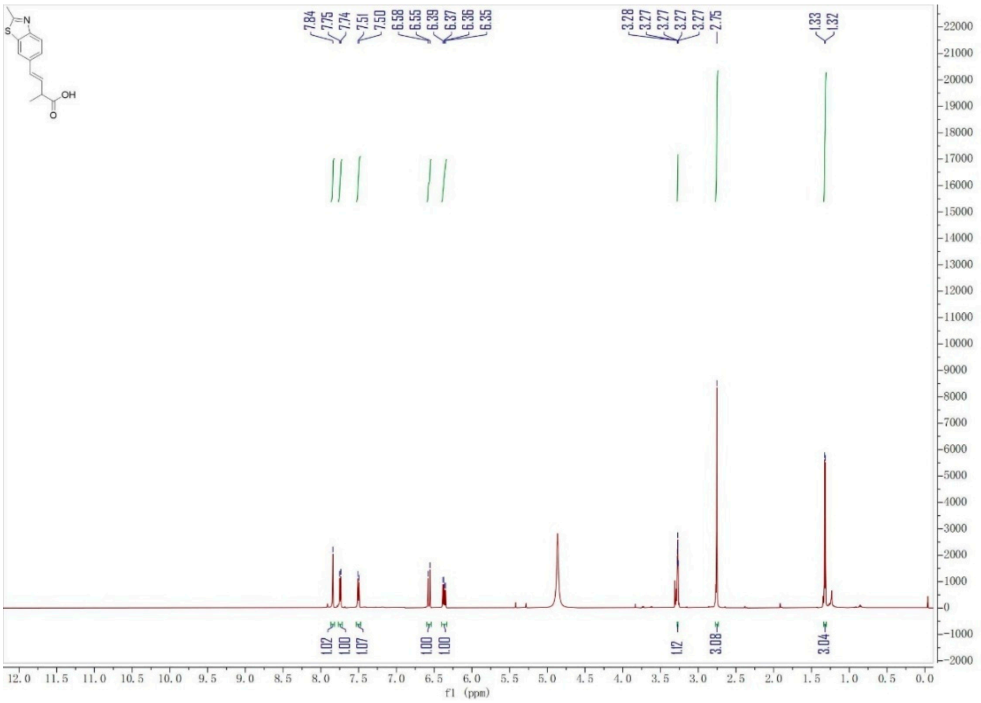

Figure S4 ^{1}H-NMR spectrum of compound **3b**

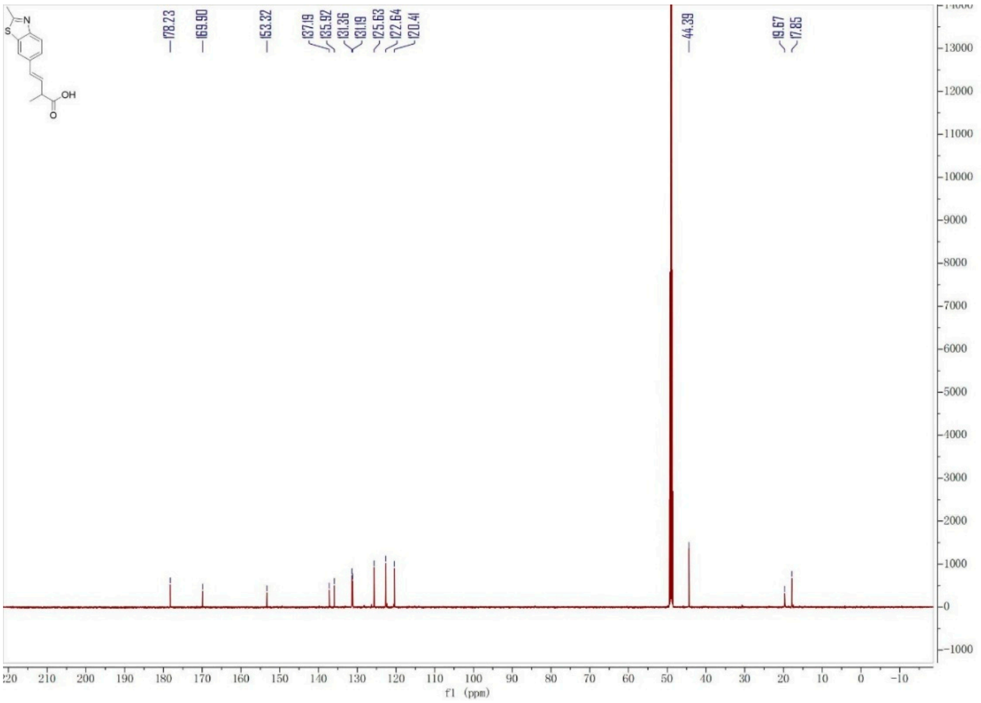

Figure S5 ^{13}C-NMR spectrum of **3b**

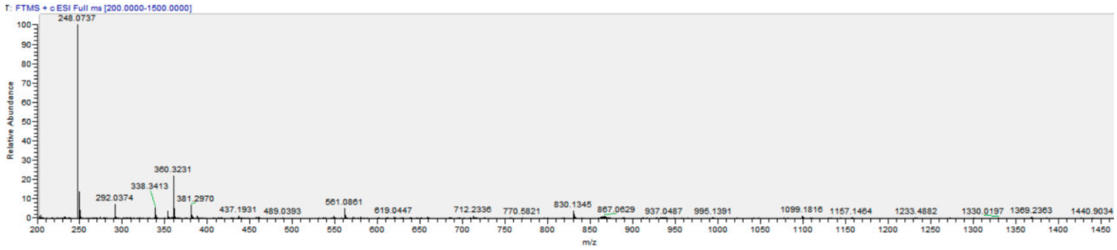

Figure S6 HR-ESI-MS spectrum of **3b**

HR-ESI-MS: m/z $[M+H]^+$ calcd for 248.0740 ($C_{13}H_{13}NO_2S$, found, 248.0737).

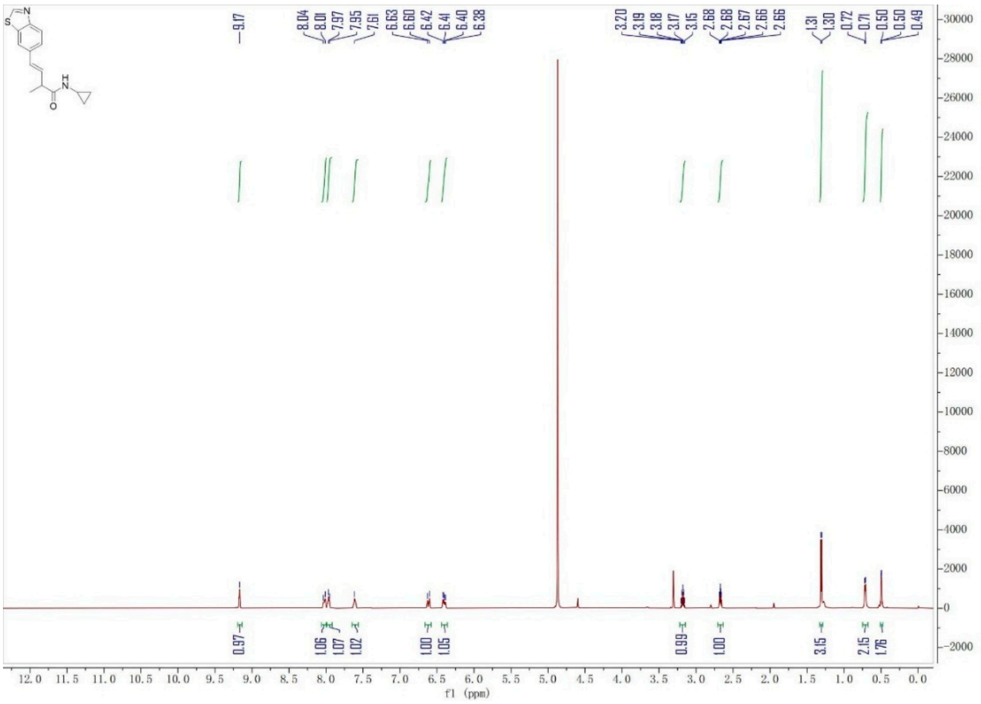

Figure S7 ^{1}H-NMR spectrum of compound **4a**

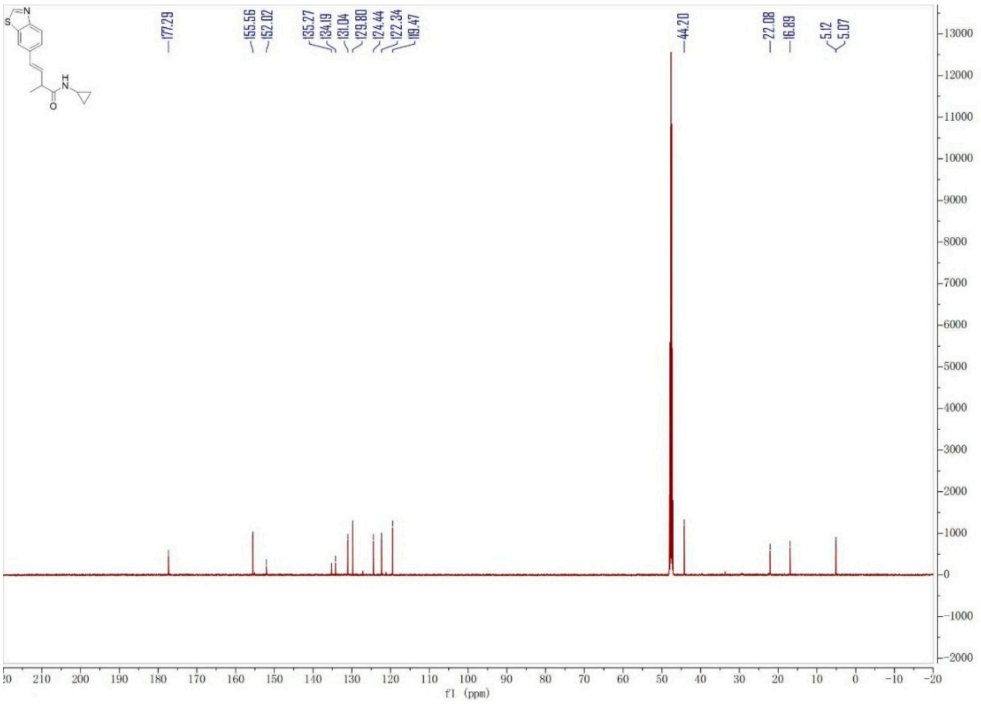

Figure S8 ^{13}C-NMR spectrum of **4a**

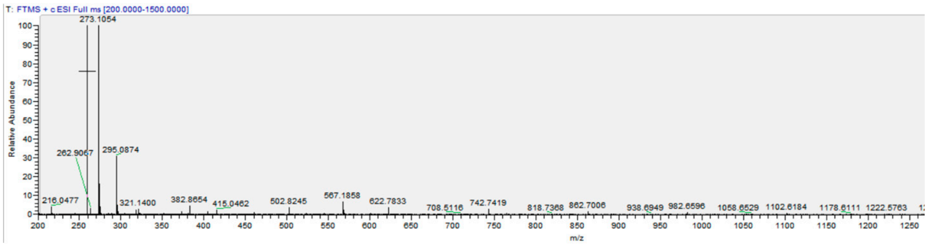

Figure S9 HR-ESI-MS spectrum of **4a**

HR-ESI-MS: m/z $[M+H]^+$ calcd for 273.1056 ($C_{15}H_{16}N_2OS$, found, 273.1054).

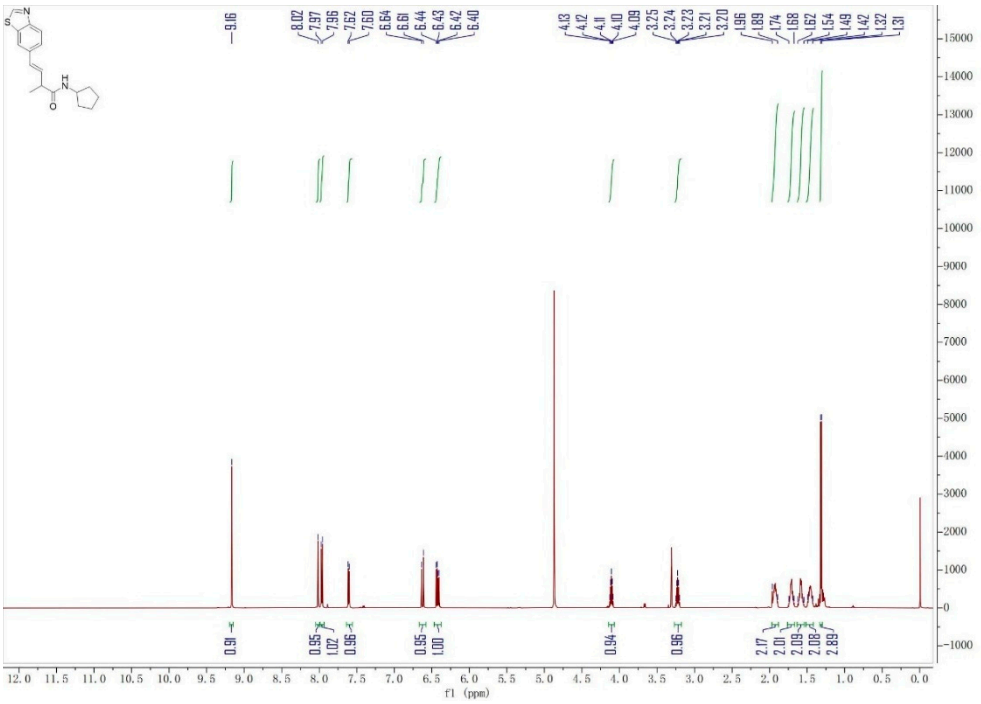

Figure S10 ^{1}H-NMR spectrum of compound **4b**

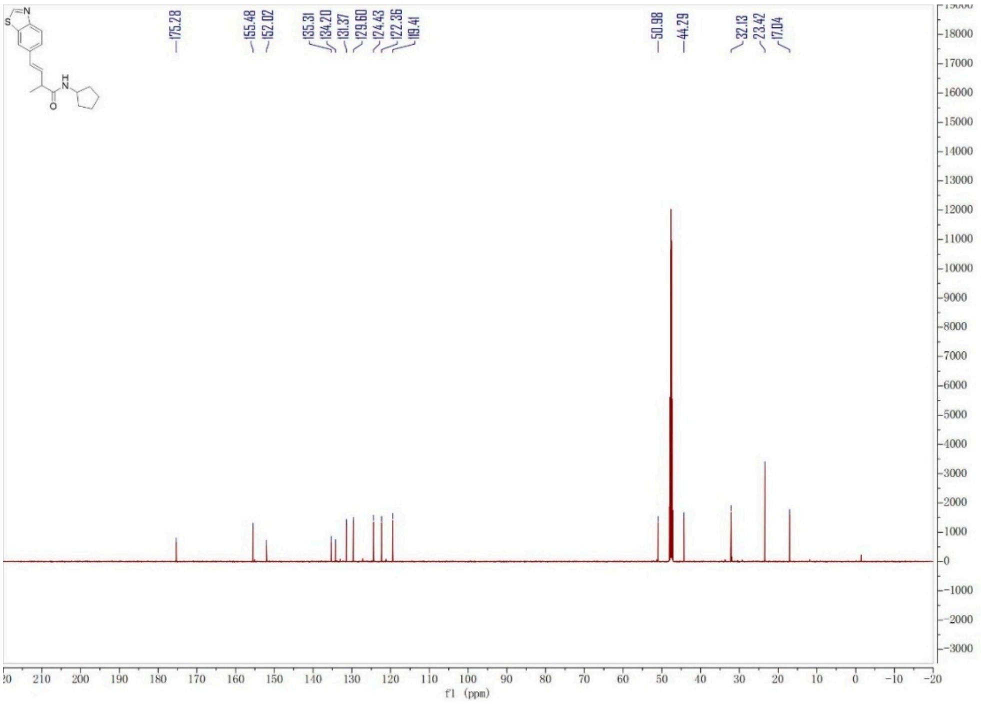

Figure S11 ^{13}C-NMR spectrum of **4b**

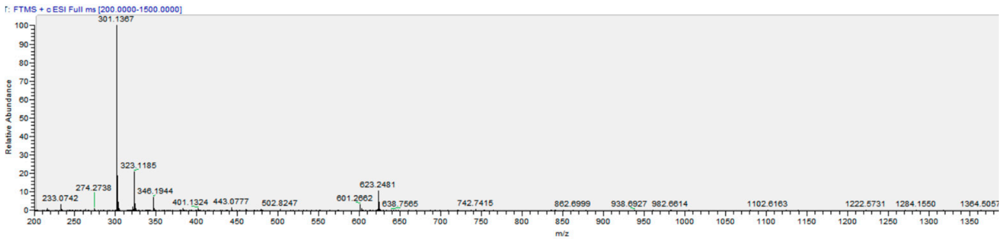

Figure S12 HR-ESI-MS spectrum of **4b**

HR-ESI-MS: m/z $[M+H]^+$ calcd for 301.1369 ($C_{17}H_{20}N_2OS$, found, 301.1367).

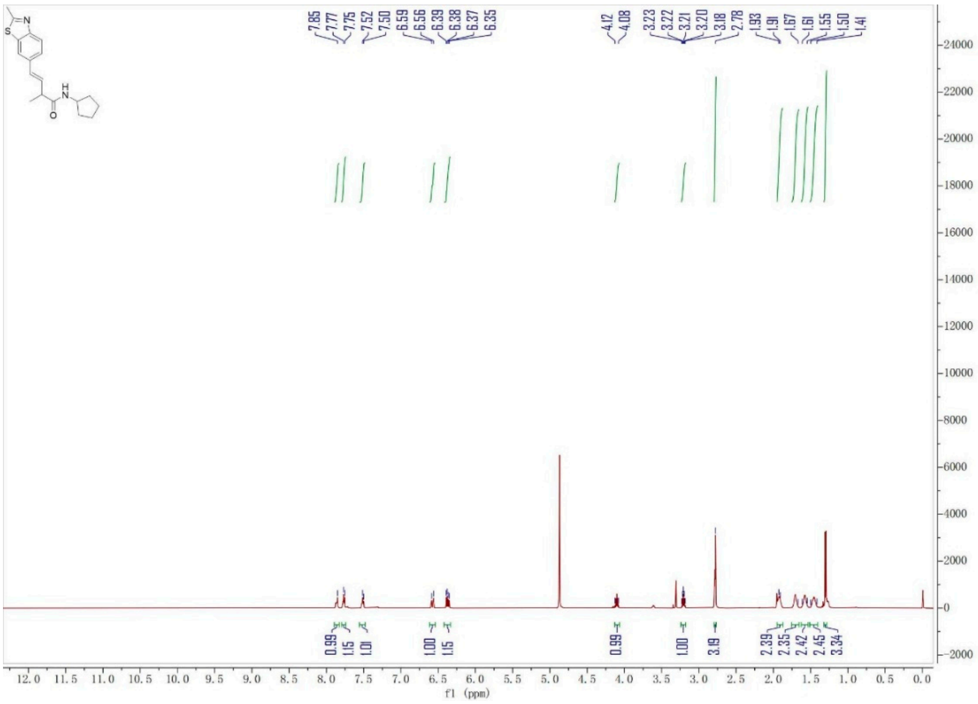

Figure S13 ^{1}H-NMR spectrum of compound **4c**

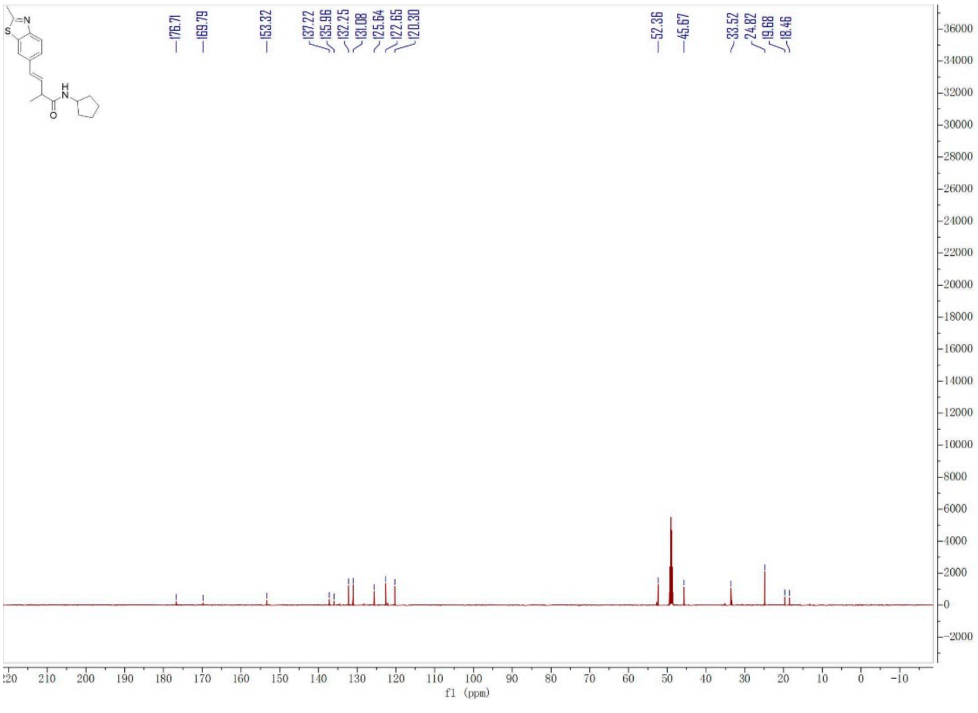

Figure S14 ^{13}C-NMR spectrum of **4c**

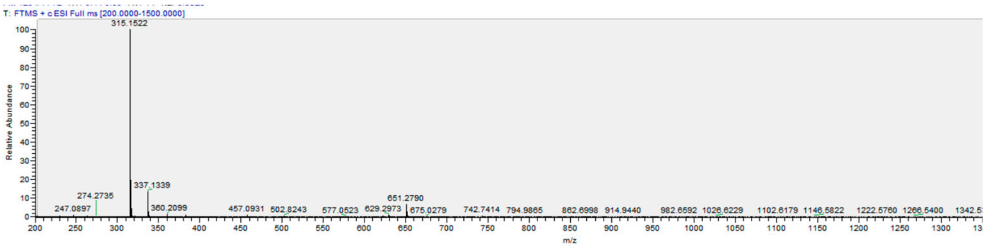

Figure S15 HR-ESI-MS spectrum of **4c**

HR-ESI-MS: m/z $[M+H]^+$ calcd for 315.1526 ($C_{18}H_{22}N_2OS$, found, 315.1522).

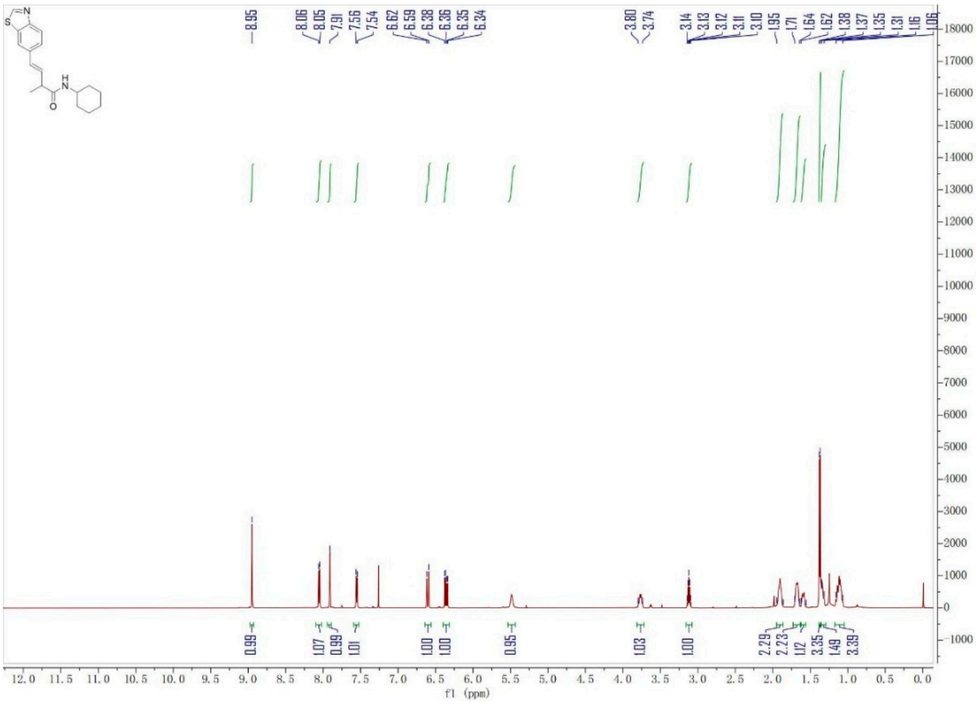

Figure S16 ^{1}H-NMR spectrum of compound **4d**

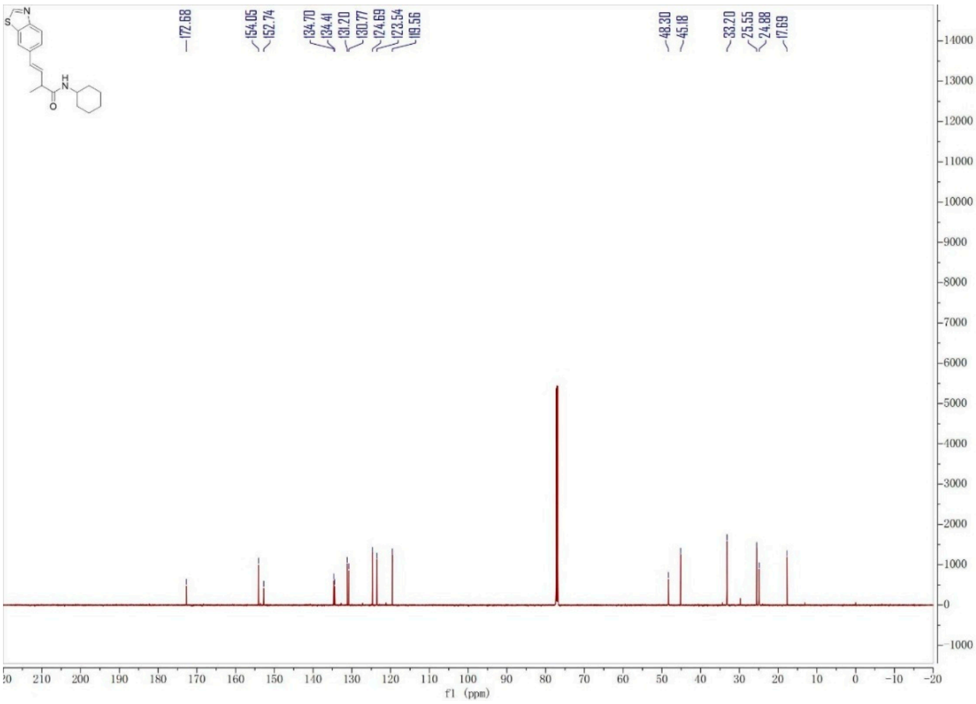

Figure S17 ^{13}C-NMR spectrum of **4d**

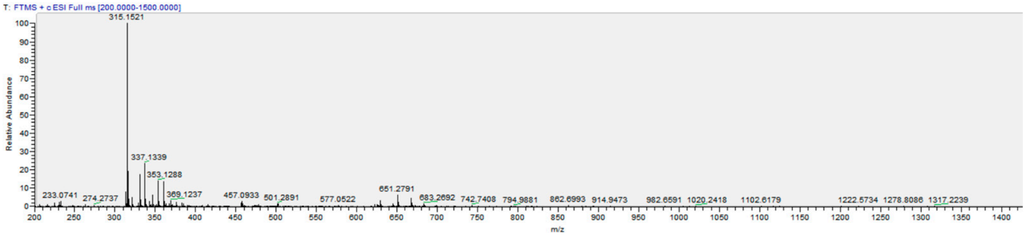

Figure S18 HR-ESI-MS spectrum of **4d**

HR-ESI-MS: m/z $[M+H]^+$ calcd for 315.1526 ($C_{18}H_{22}N_2OS$, found, 315.1521).

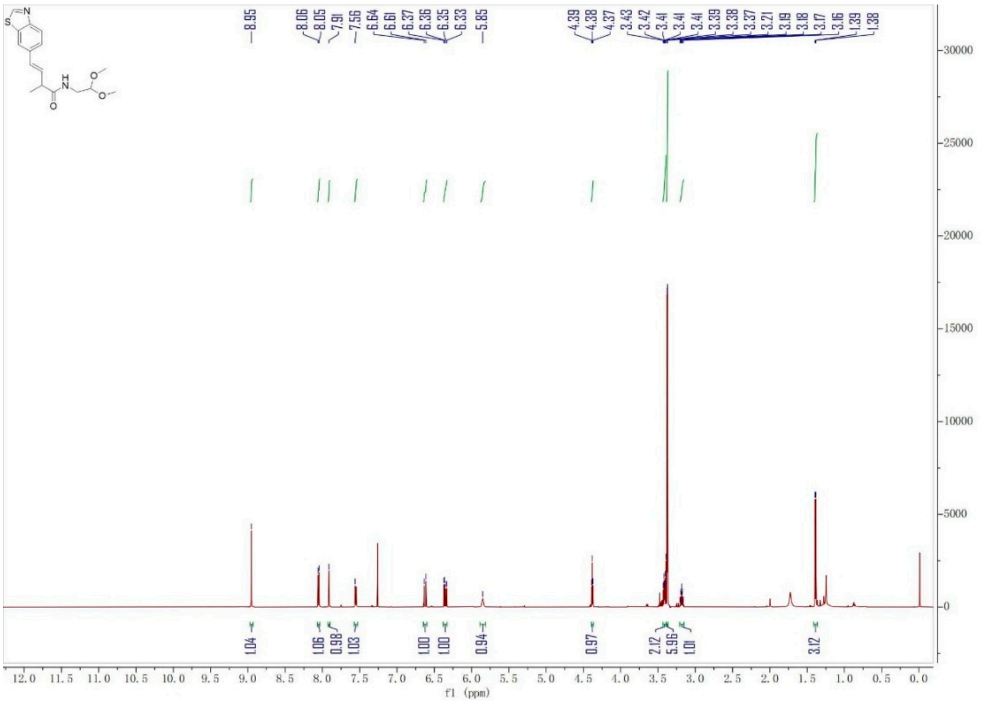

Figure S19 ^{1}H-NMR spectrum of compound **4e**

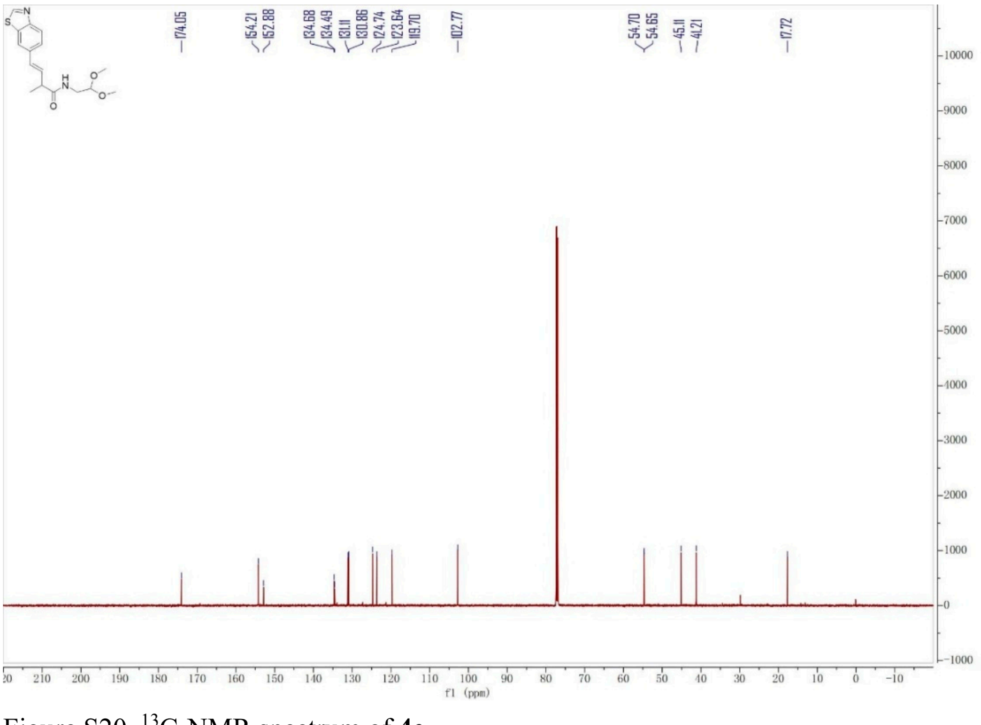

Figure S20 ^{13}C-NMR spectrum of **4e**

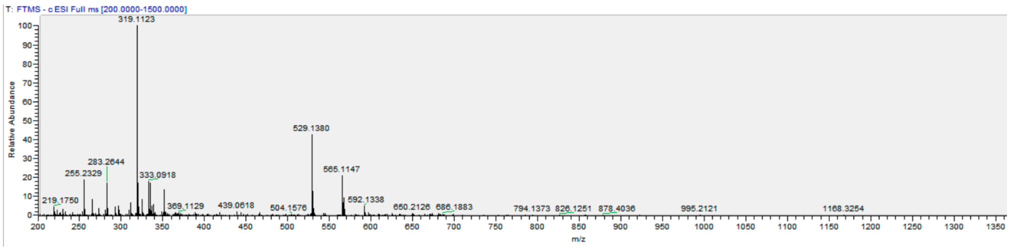

Figure S21 HR-ESI-MS spectrum of **4e**

HR-ESI-MS: m/z $[M-H]^-$ calcd for 319.1122 ($C_{16}H_{20}N_2O_3S$, found, 319.1123).

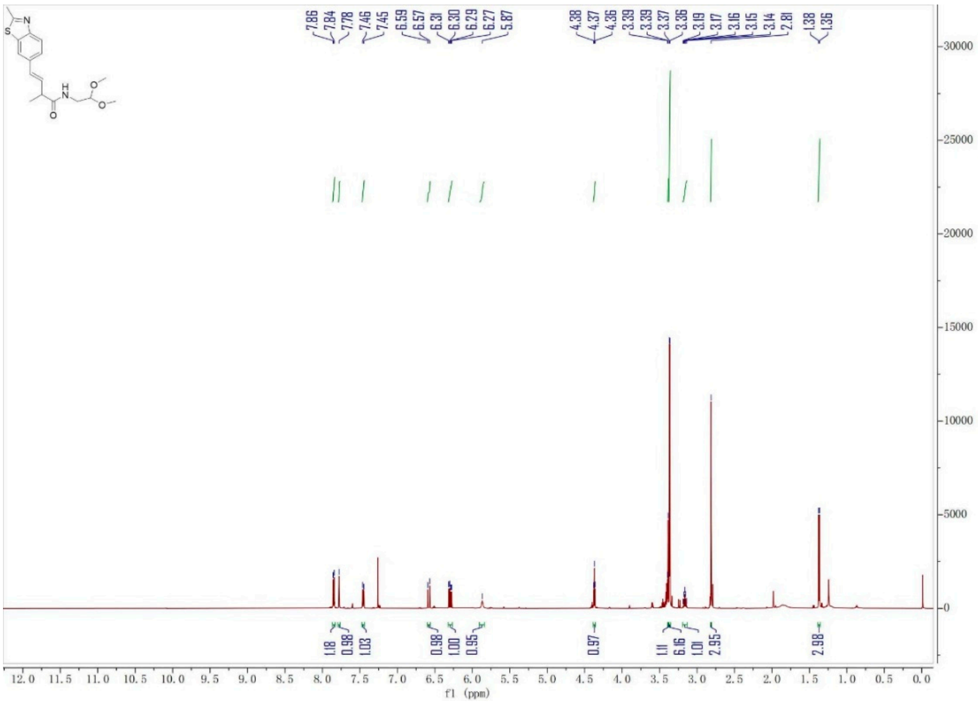

Figure S22 ^{1}H-NMR spectrum of compound **4f**

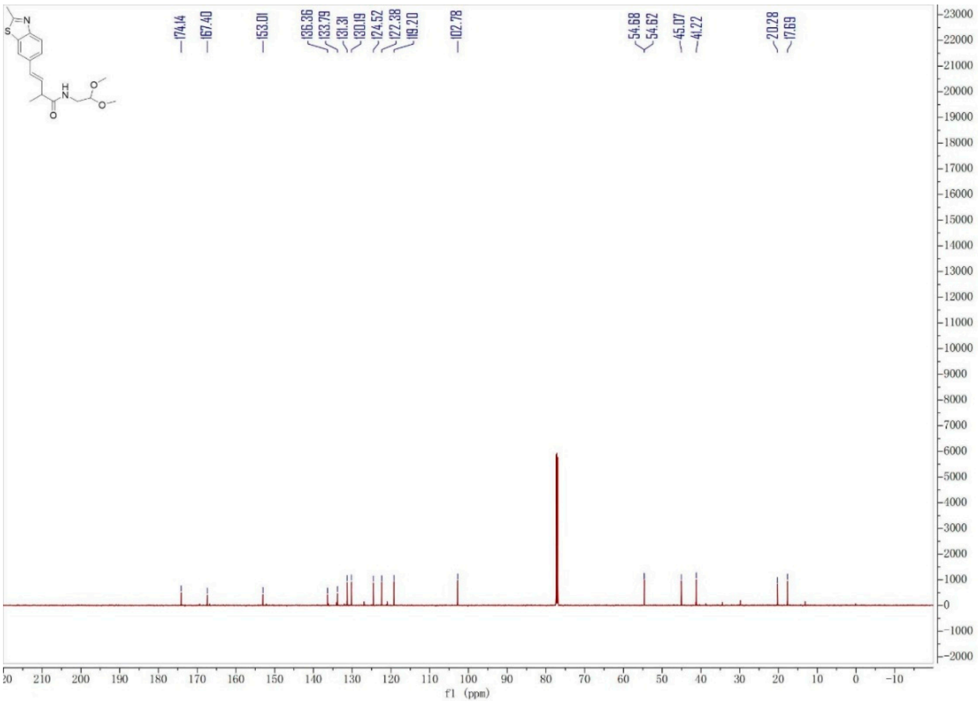

Figure S23 ^{13}C-NMR spectrum of **4f**

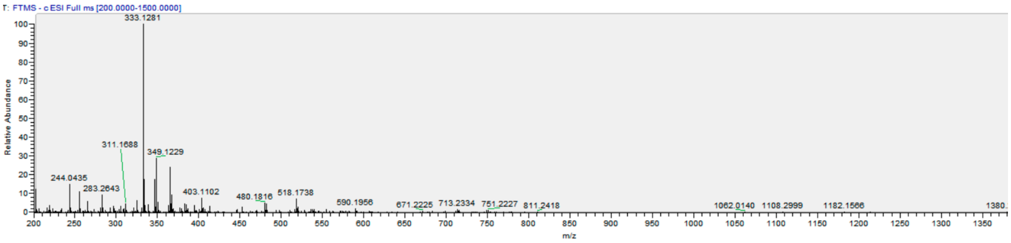

Figure S24 HR-ESI-MS spectrum of **4f**

HR-ESI-MS: m/z [M-H]$^-$ calcd for 333.1278 ($C_{17}H_{22}N_2O_3S$, found, 333.1281).

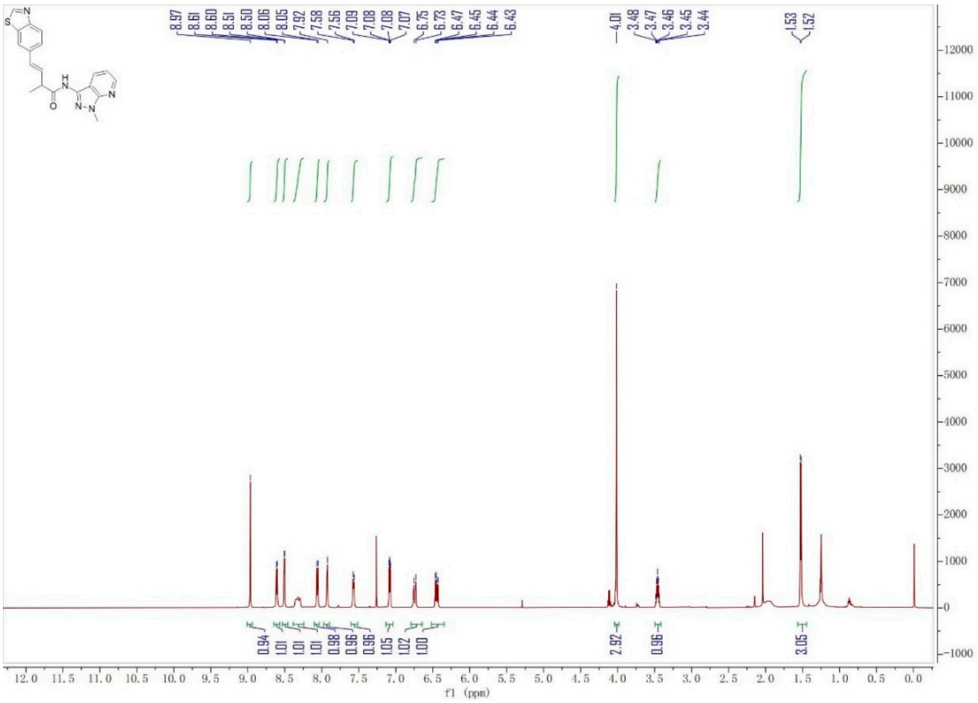

Figure S25 ^{1}H-NMR spectrum of compound **4g**

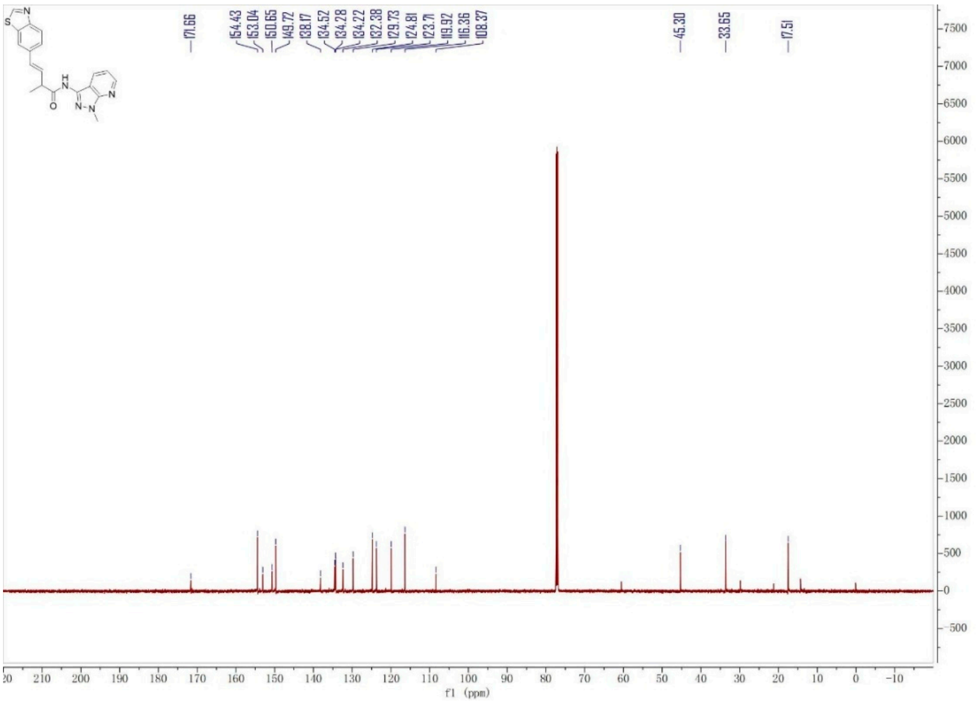

Figure S26 ^{13}C-NMR spectrum of compound **4g**

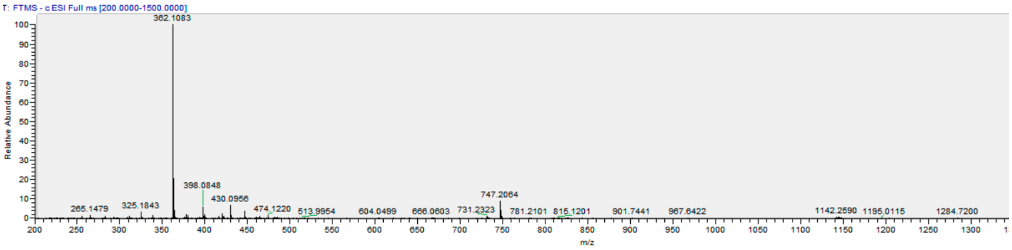

Figure S27 HR-ESI-MS spectrum of **4g**

HR-ESI-MS: m/z [M-H]- calcd for 362.1081 ($C_{19}H_{17}N_5OS$, found, 362.1083).

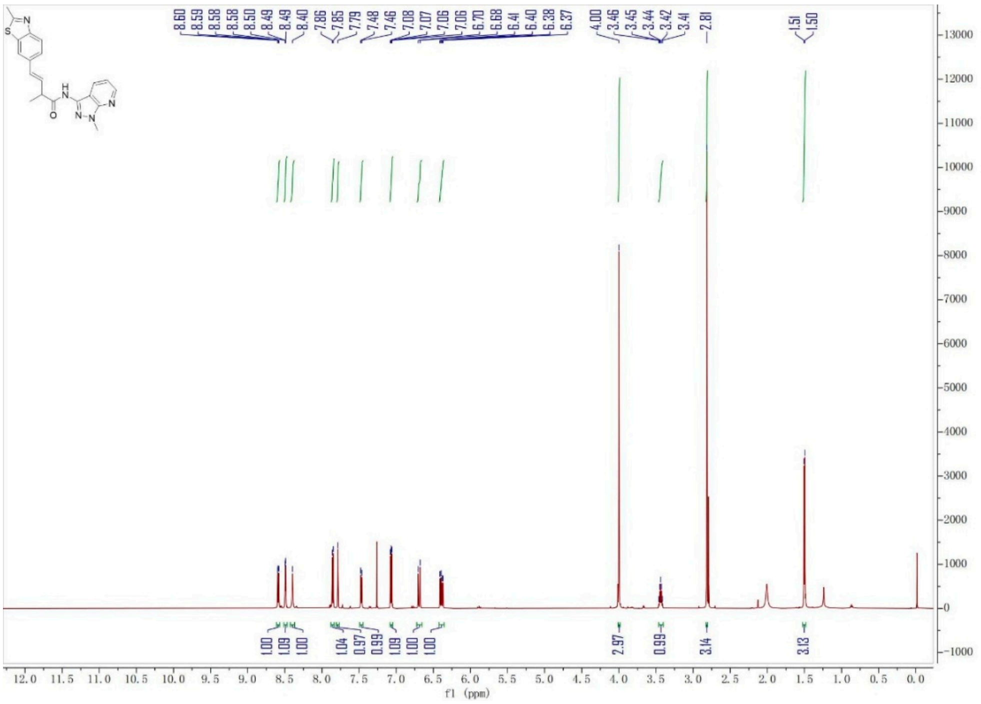

Figure S28 ^{1}H-NMR spectrum of compound **4h**

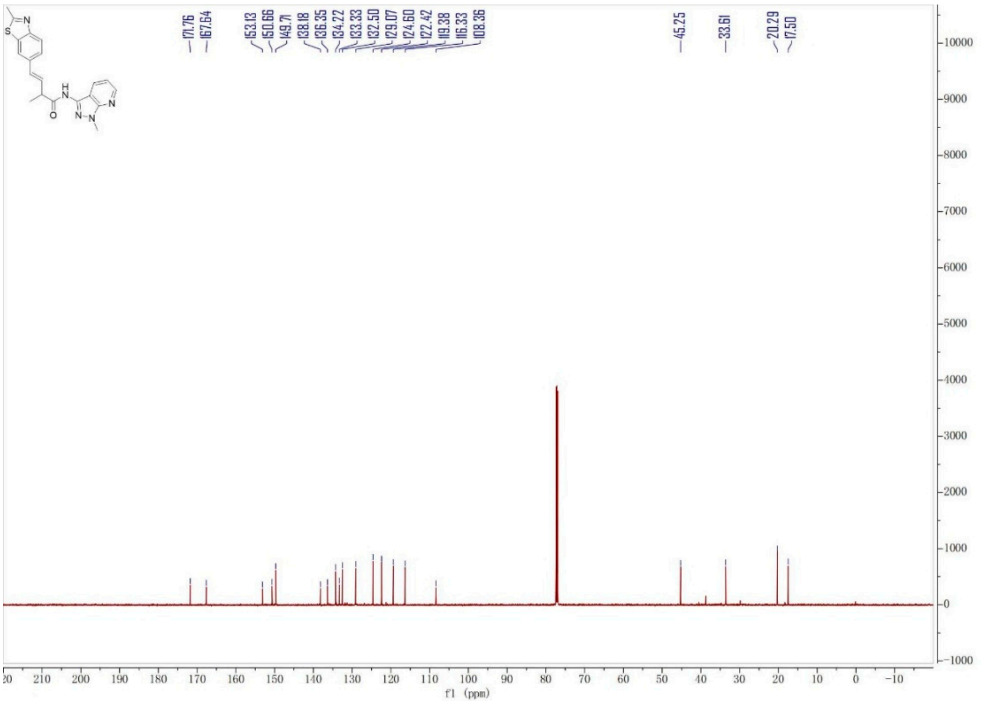

Figure S29 ^{13}C-NMR spectrum of compound **4h**

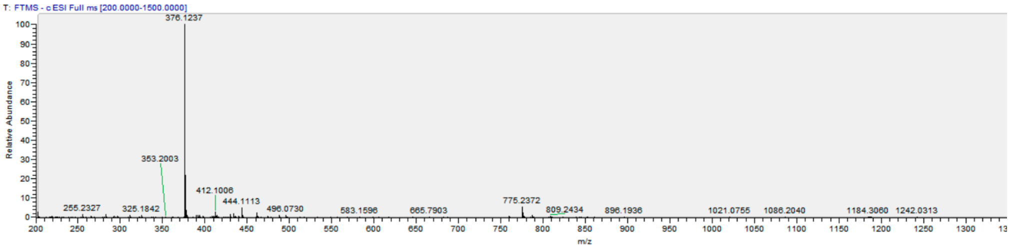

Figure S30 HR-ESI-MS spectrum of **4h**

HR-ESI-MS: m/z [M-H]$^-$ calcd for 376.1238 ($C_{20}H_{19}N_5OS$, found, 376.1237).

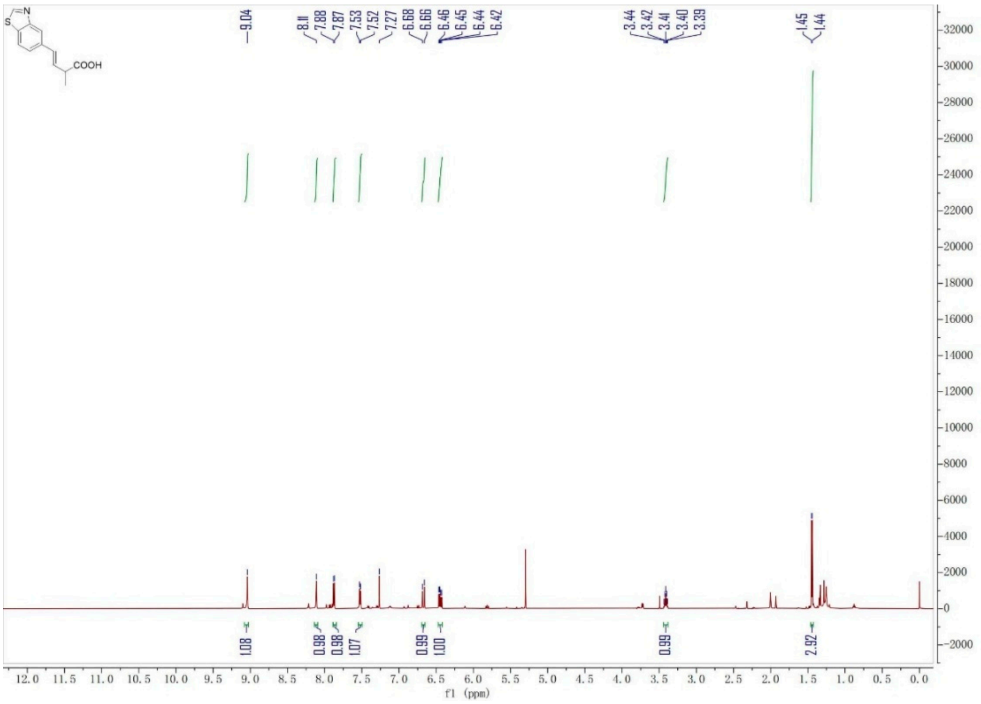

Figure S31 ^{1}H-NMR spectrum of compound **6a**

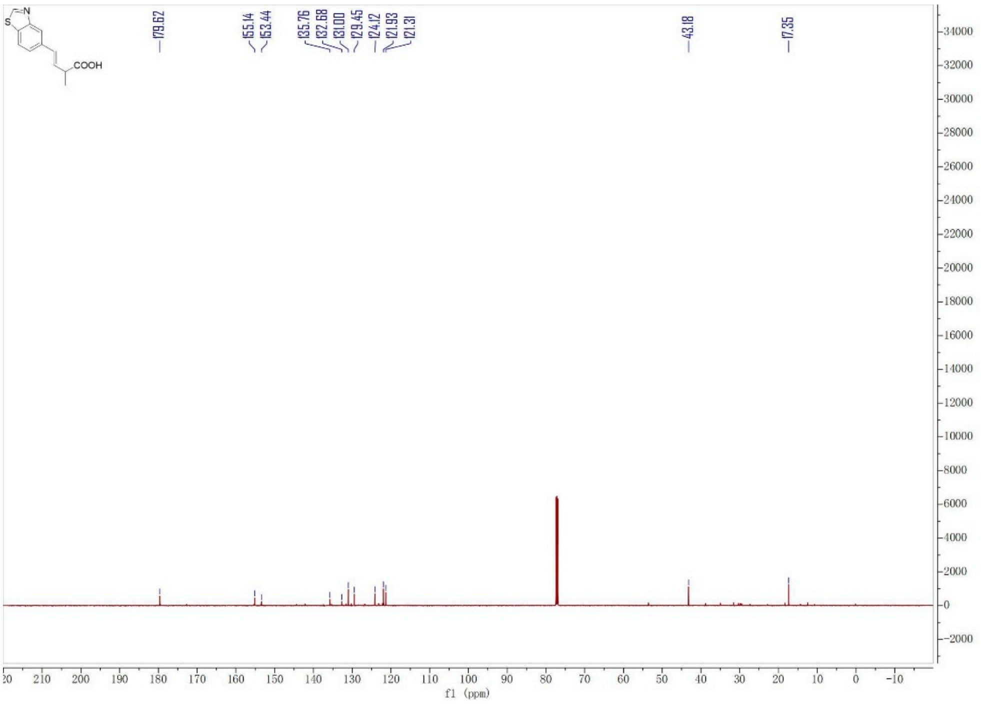

Figure S32 ^{13}C-NMR spectrum of **6a**

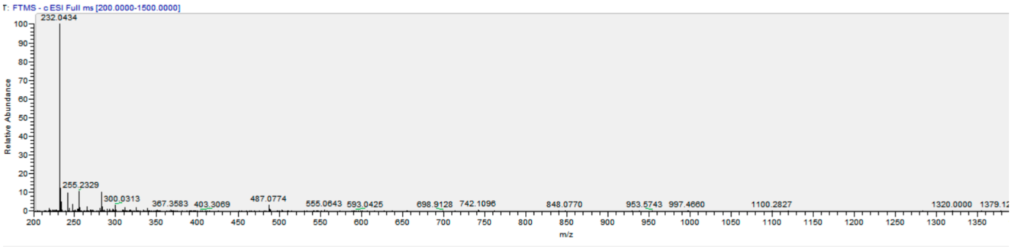

Figure S33 HR-ESI-MS spectrum of **6a**

HR-ESI-MS: m/z [M-H]$^-$ calcd for 232.0438 ($C_{12}H_{11}NO_2S$, found, 232.0434).

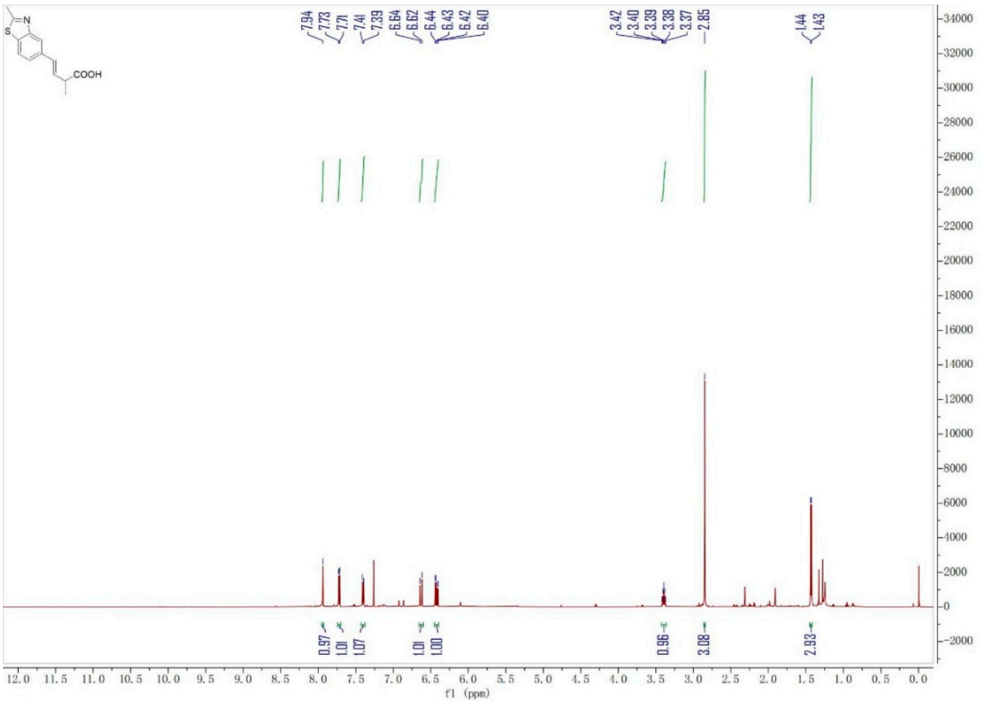

Figure S34 ^{1}H-NMR spectrum of compound **6b**

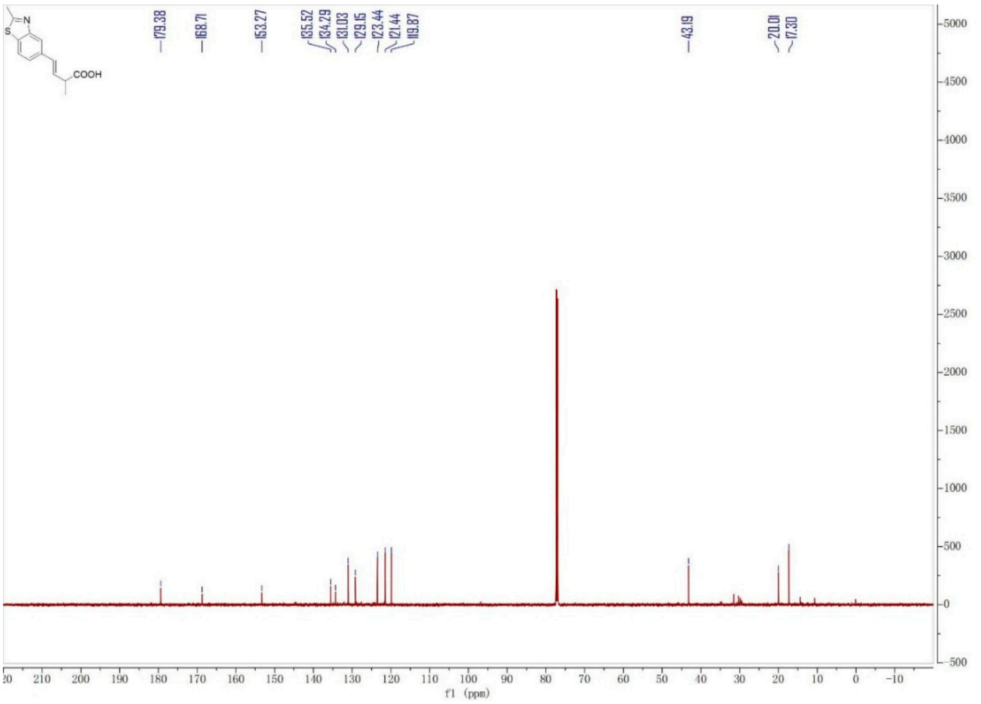

Figure S35 ^{13}C-NMR spectrum of **6b**

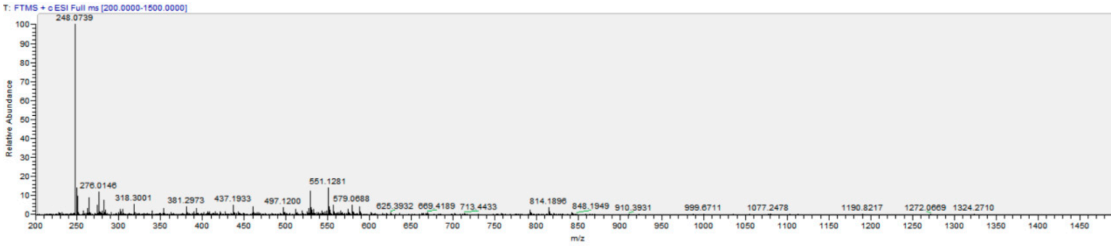

Figure S36 HR-ESI-MS spectrum of **6b**

HR-ESI-MS: m/z $[M+H]^+$ calcd for 248.0740 ($C_{13}H_{13}NO_2S$, found, 248.0739).

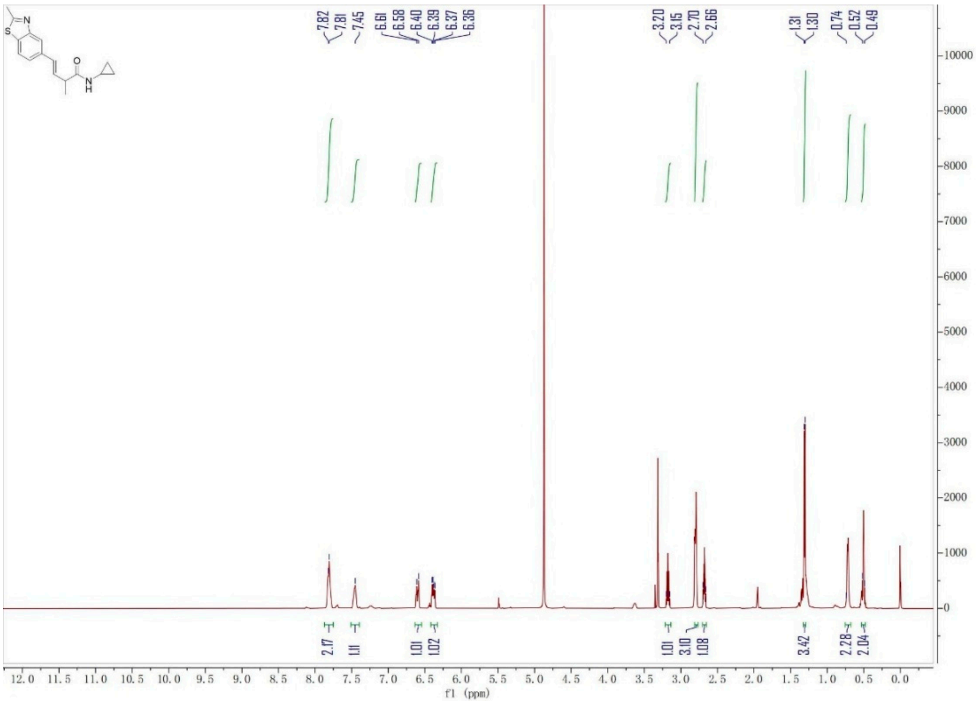

Figure S37 ^{1}H-NMR spectrum of compound **7a**

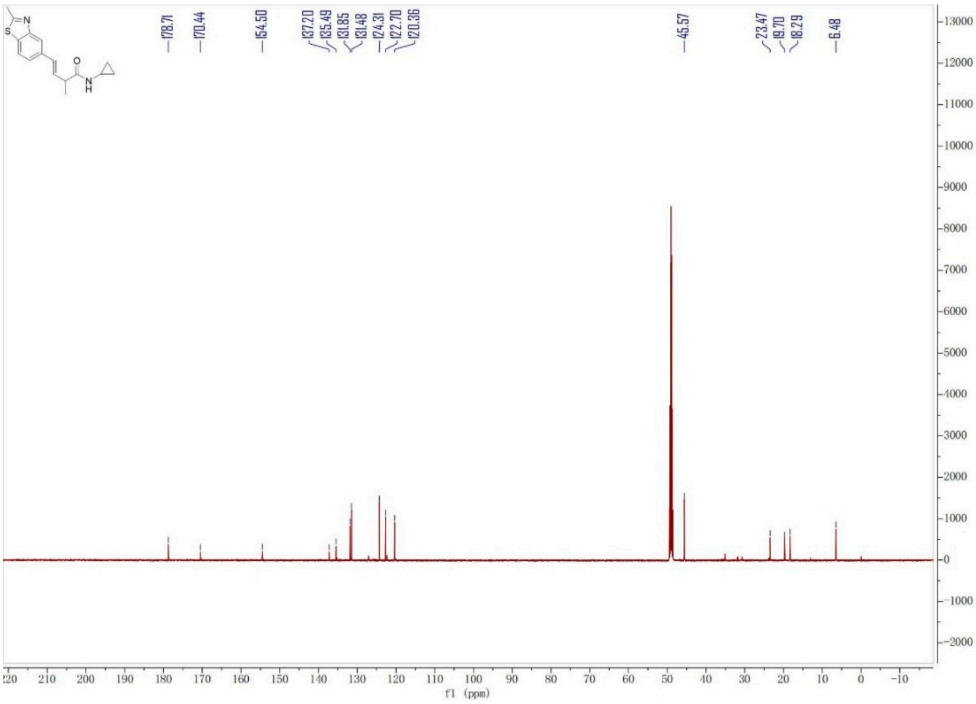

Figure S38 ^{13}C-NMR spectrum of **7a**

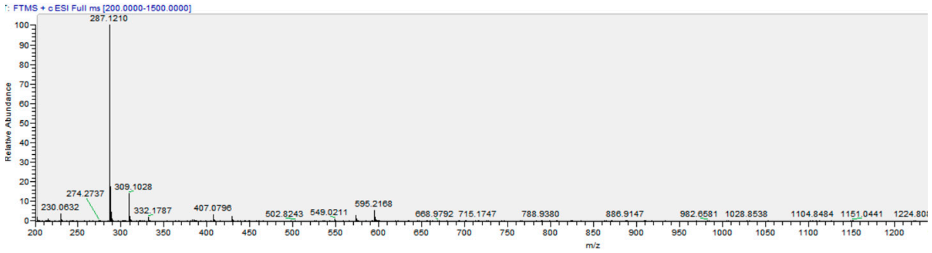

Figure S39 HR-ESI-MS spectrum of **7a**

HR-ESI-MS: m/z $[M+H]^+$ calcd for 287.1213 ($C_{16}H_{18}N_2OS$, found, 287.1210).

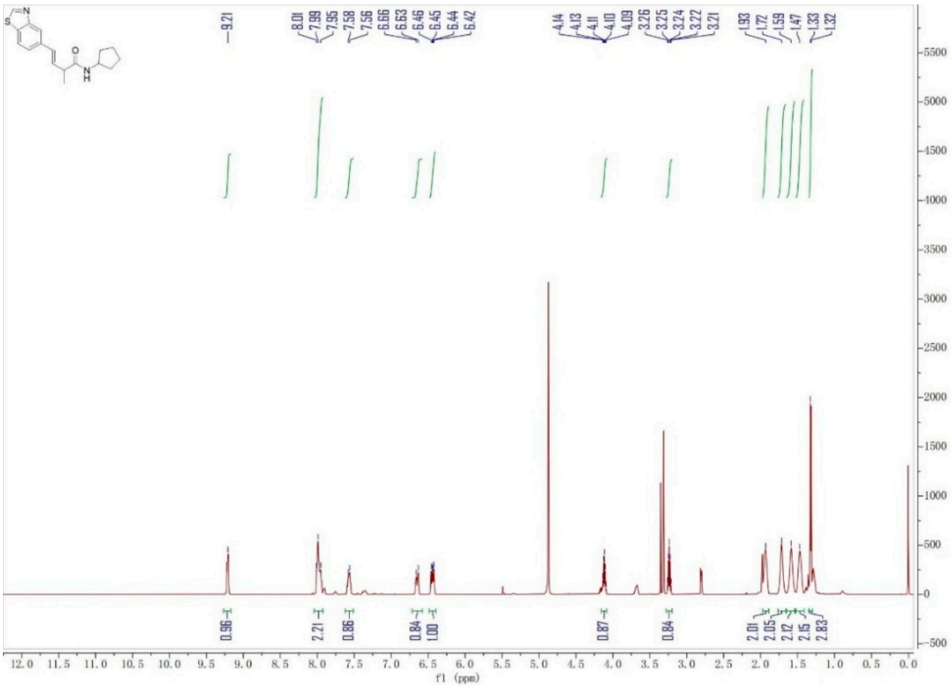

Figure S40 ^{1}H-NMR spectrum of compound **7b**

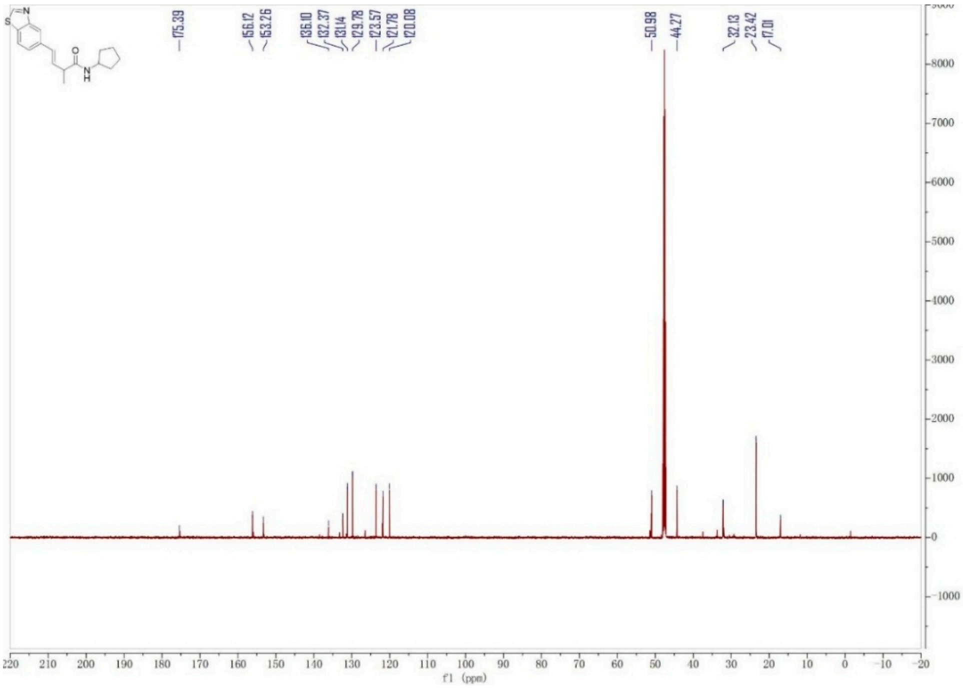

Figure S41 ^{13}C-NMR spectrum of **7b**

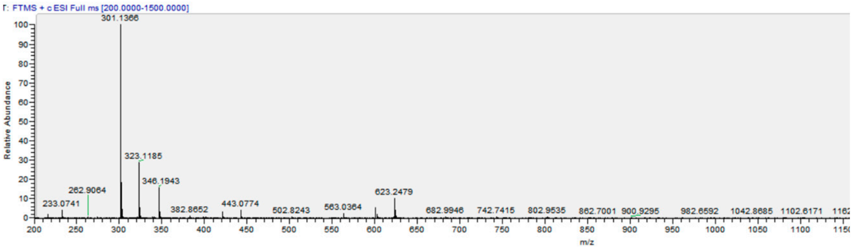

Figure S42 HR-ESI-MS spectrum of **7b**

HR-ESI-MS: m/z $[M+H]^+$ calcd for 301.1369 ($C_{17}H_{20}N_2OS$, found, 301.1366).

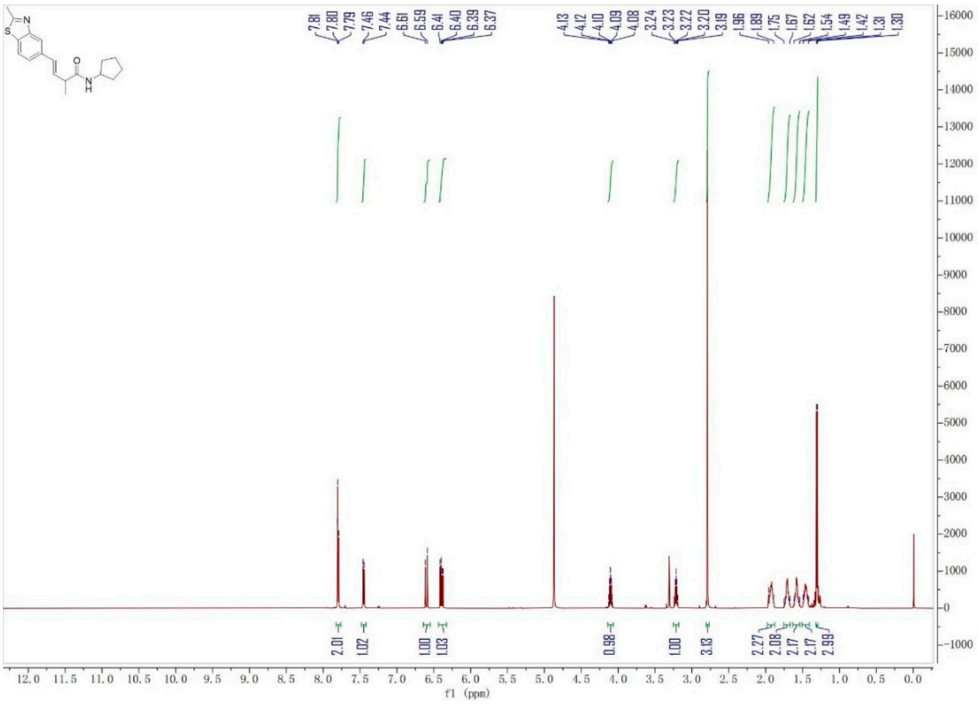

Figure S43 ^{1}H-NMR spectrum of compound **7c**

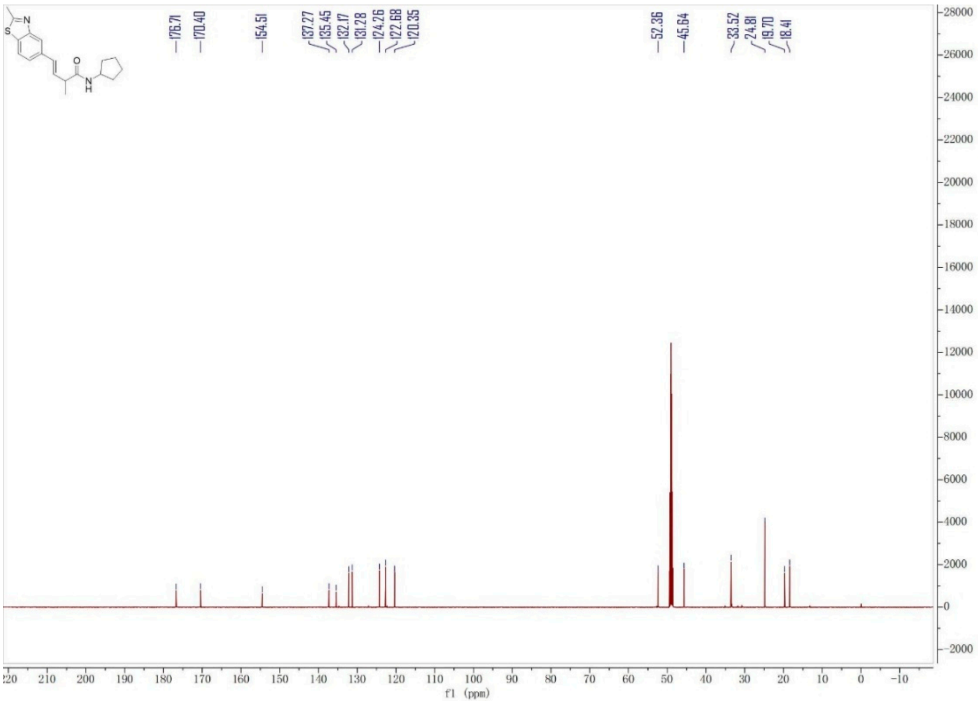

Figure S44 ^{13}C-NMR spectrum of **7c**

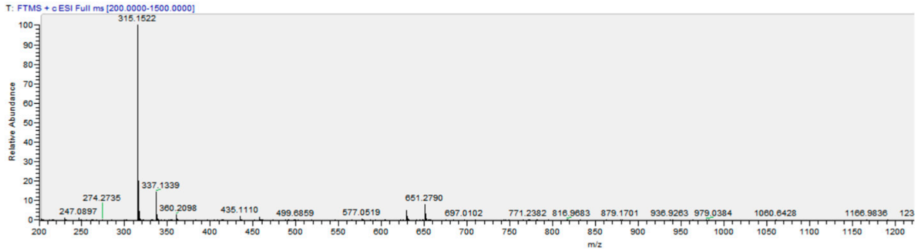

Figure S45 HR-ESI-MS spectrum of **7c**

HR-ESI-MS: m/z $[M+H]^+$ calcd for 315.1523 ($C_{18}H_{22}N_2OS$, found, 315.1522).

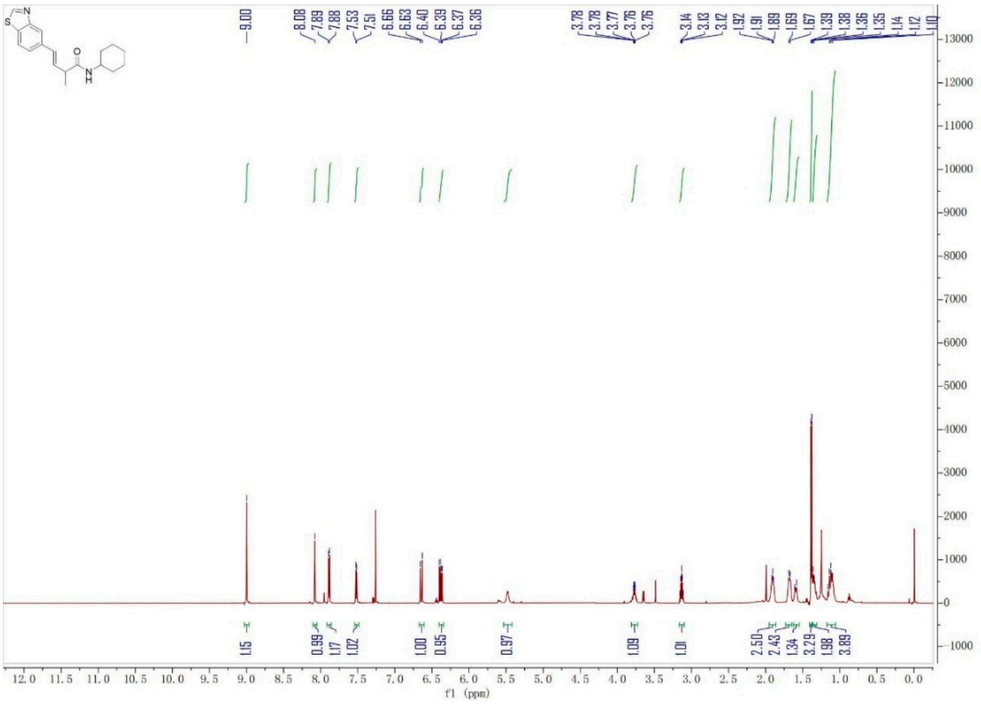

Figure S46 ^{1}H-NMR spectrum of compound **7d**

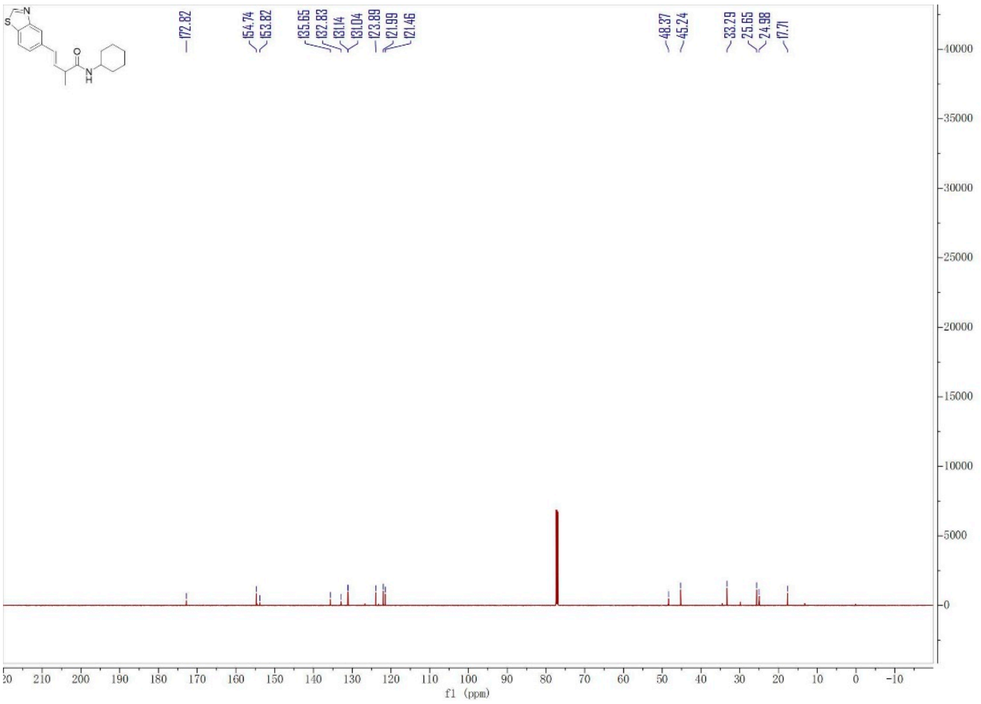

Figure S47 ^{13}C-NMR spectrum of **7d**

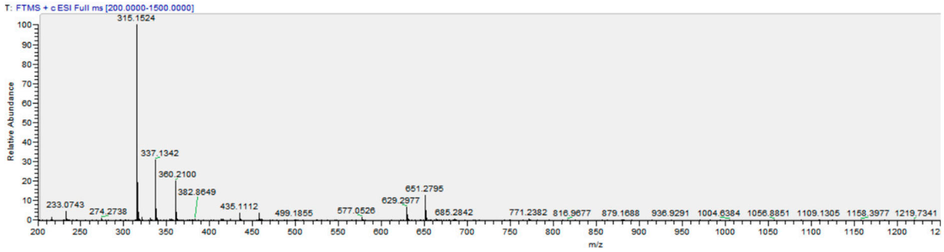

Figure S48 HR-ESI-MS spectrum of **7d**

HR-ESI-MS: m/z [M+H]+ calcd for 315.1526 ($C_{18}H_{22}N_2OS$, found, 315.1524).

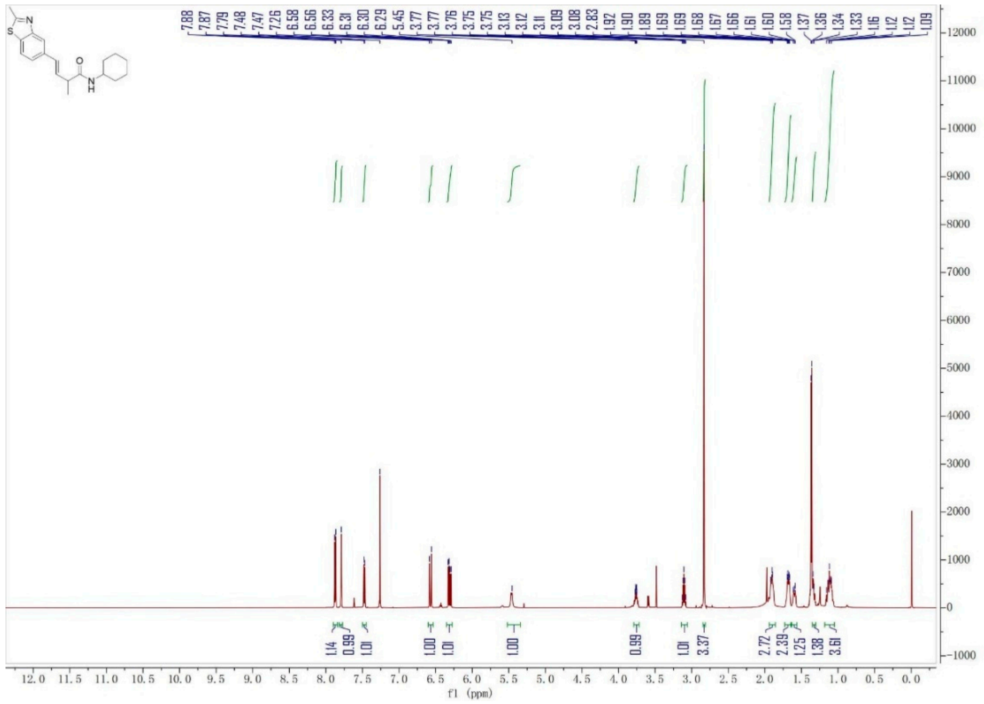

Figure S49 ^{1}H-NMR spectrum of compound **7e**

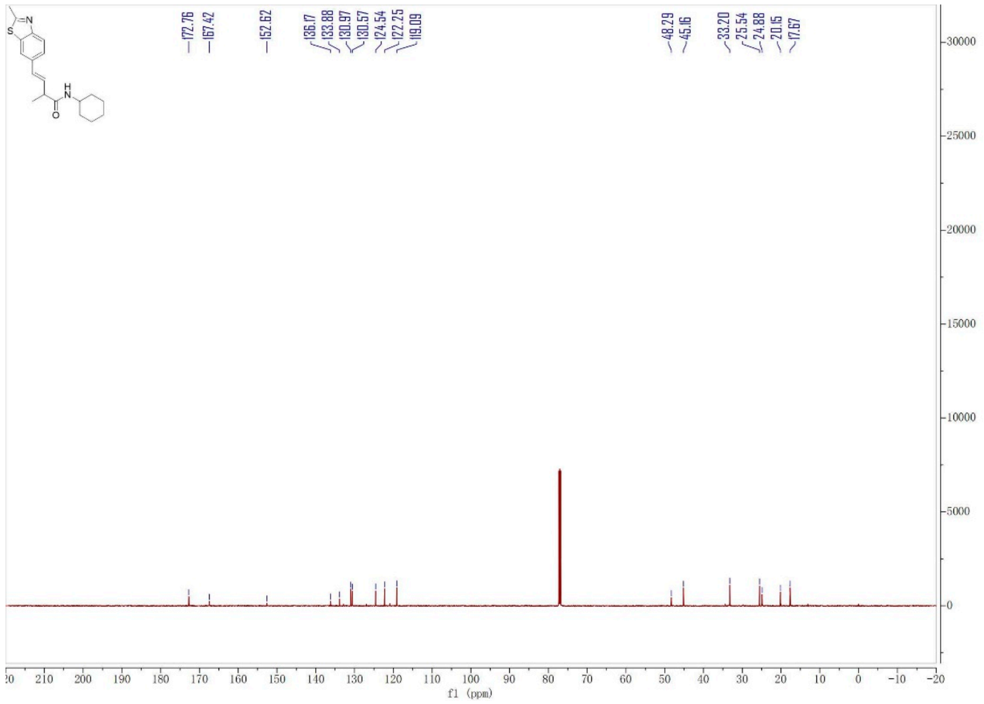

Figure S50 ^{13}C-NMR spectrum of **7e**

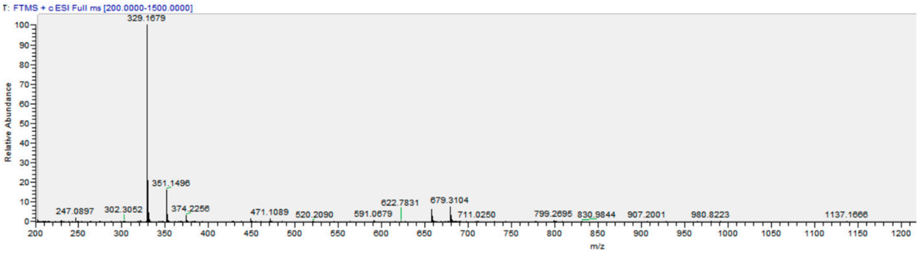

Figure S51 HR-ESI-MS spectrum of **7e**

HR-ESI-MS: m/z $[M+H]^+$ calcd for 329.1682 ($C_{19}H_{24}N_2OS$, found, 329.1679).

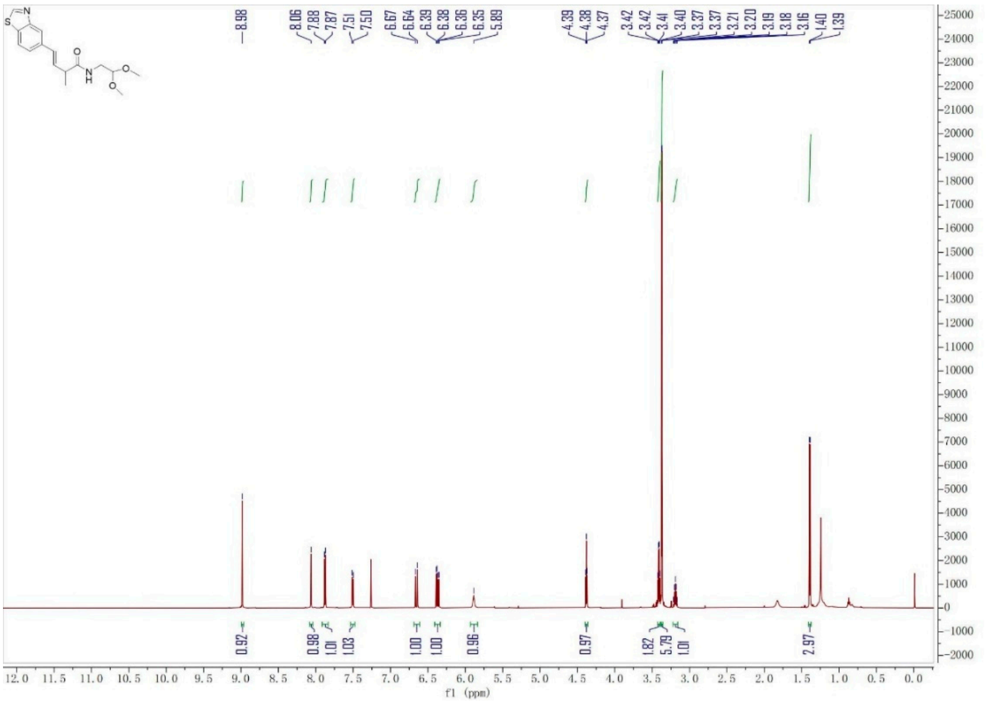

Figure S52 ^{1}H-NMR spectrum of compound **7f**

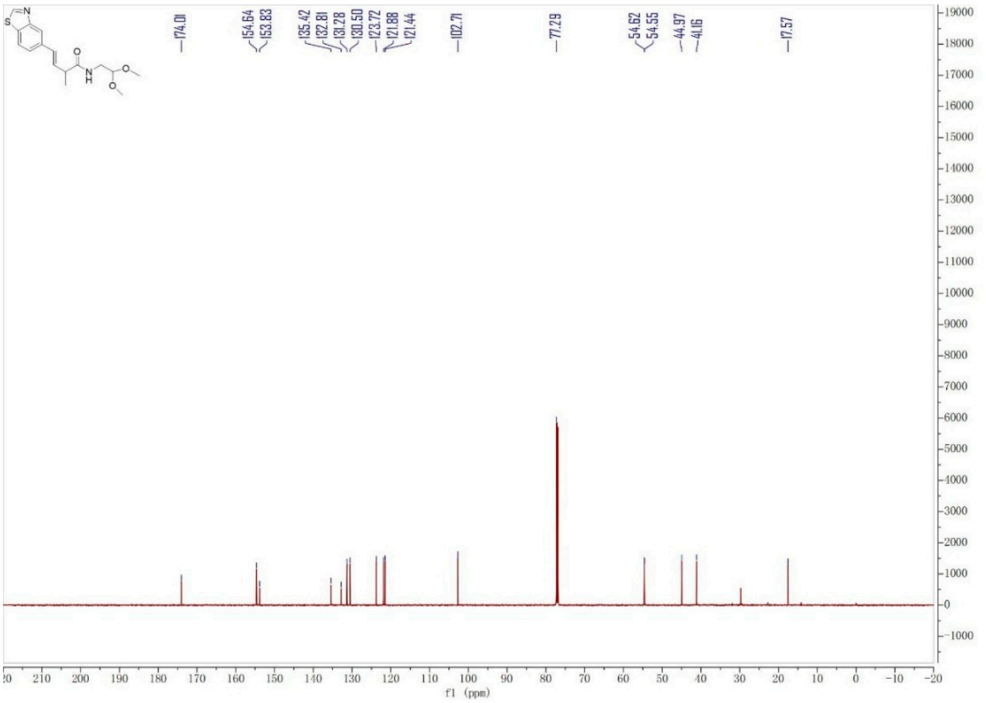

Figure S53 ^{13}C-NMR spectrum of **7f**

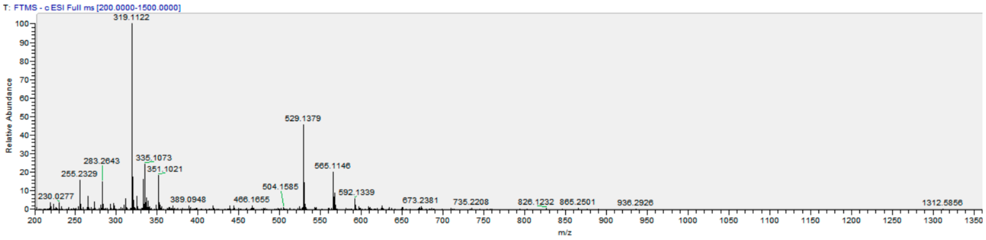

Figure S54 HR-ESI-MS spectrum of **7f**

HR-ESI-MS: m/z [M-H]$^-$ calcd for 319.1122 ($C_{16}H_{20}N_2O_3S$, found, 319.1122).

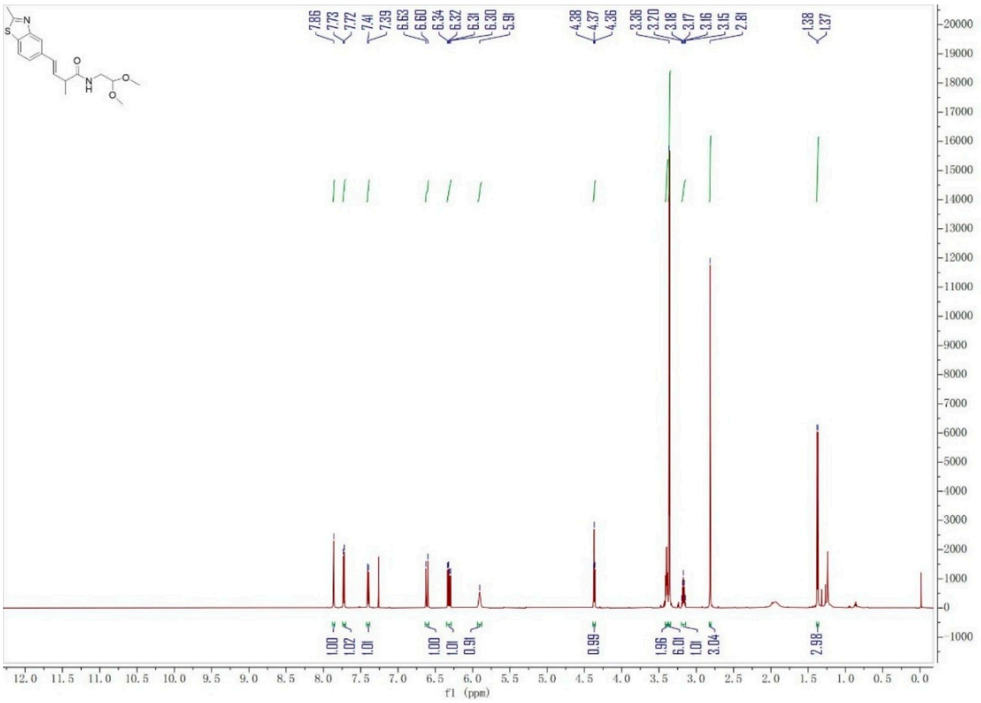

Figure S55 ^{1}H-NMR spectrum of compound **7g**

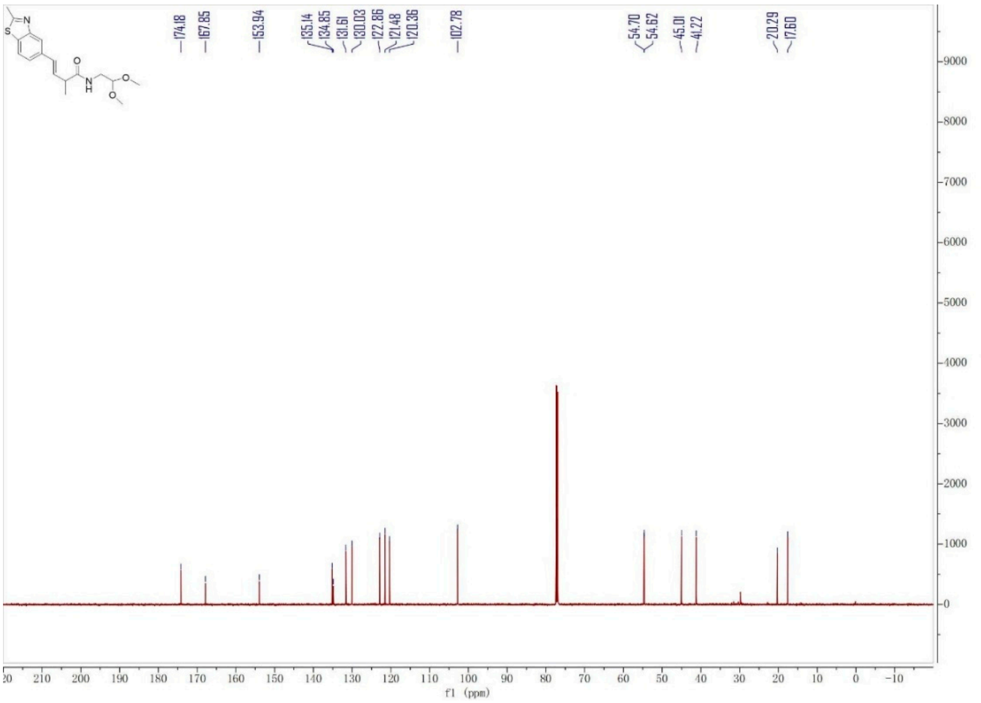

Figure S56 ^{13}C-NMR spectrum of **7g**

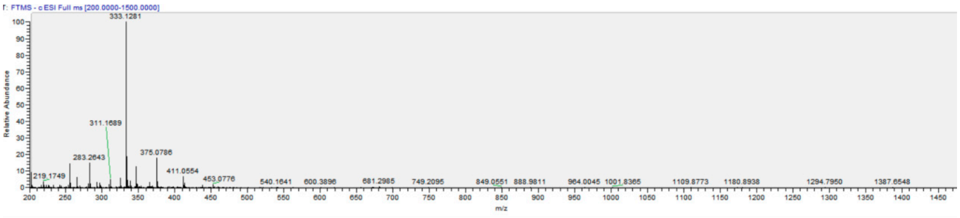

Figure S57 HR-ESI-MS spectrum of **7g**

HR-ESI-MS: m/z [M-H]- calcd for 333.1278 ($C_{19}H_{17}N_5OS$, found, 333.1281).

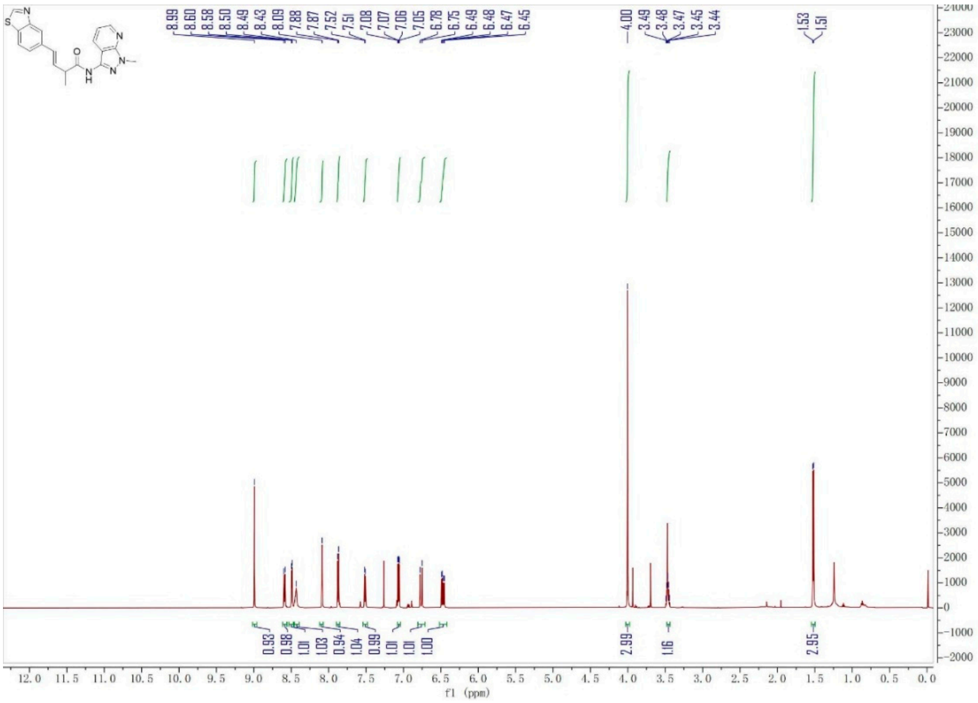

Figure S58 ^{1}H-NMR spectrum of compound **7h**

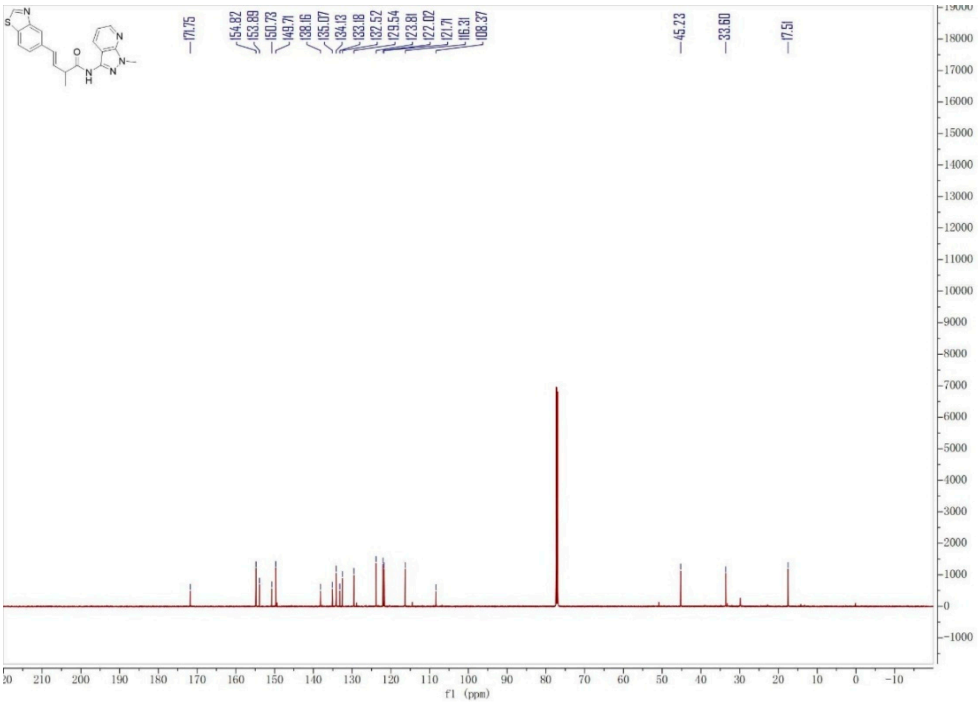

Figure S59 ^{13}C-NMR spectrum of **7h**

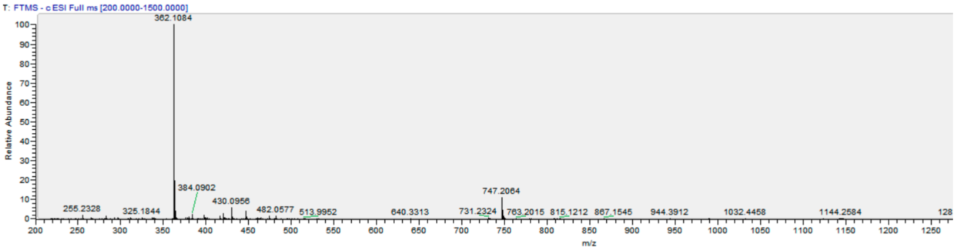

Figure S60 HR-ESI-MS spectrum of **7h**

HR-ESI-MS: m/z [M-H]$^-$ calcd for 362.1081 ($C_{19}H_{17}N_5OS$, found, 362.1084).

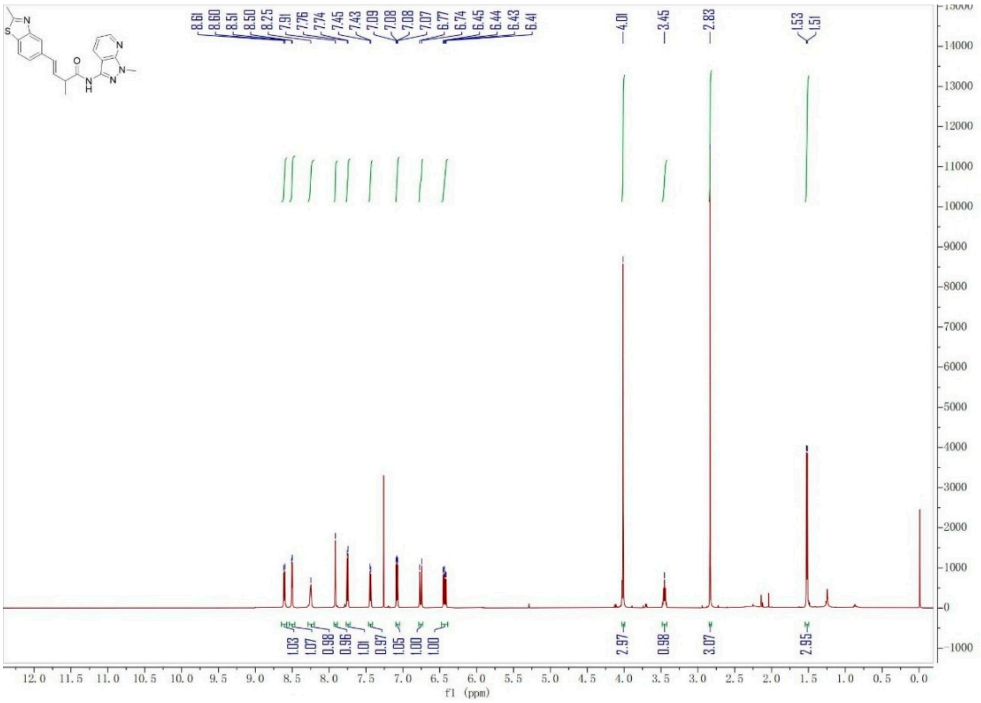

Figure S61 ^{1}H-NMR spectrum of compound **7i**

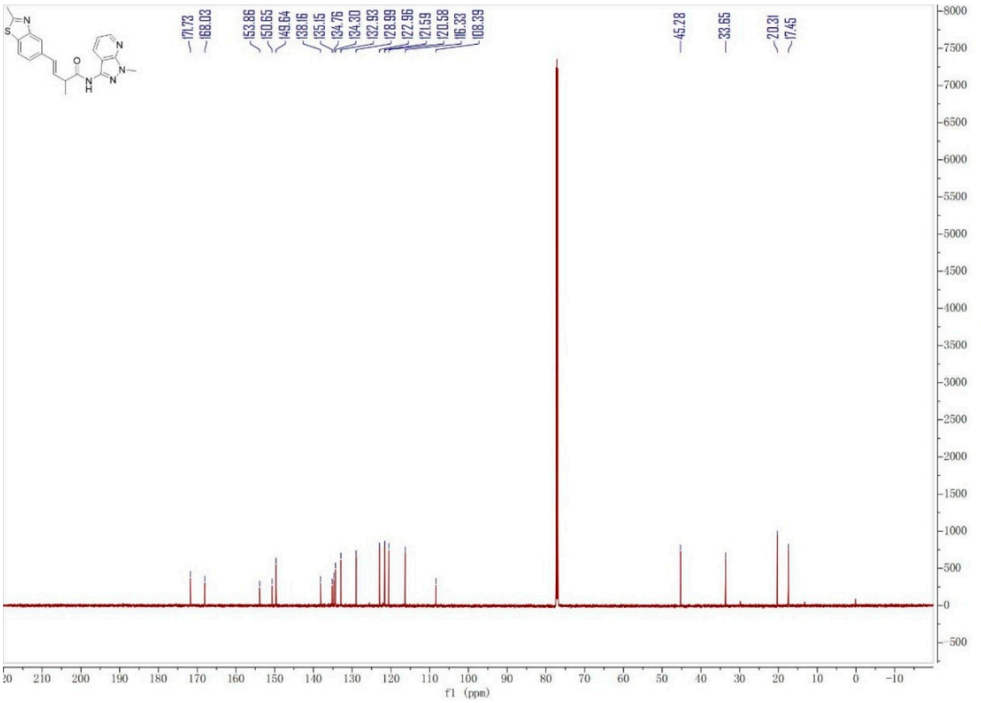

Figure S62 ^{13}C-NMR spectrum of **7i**

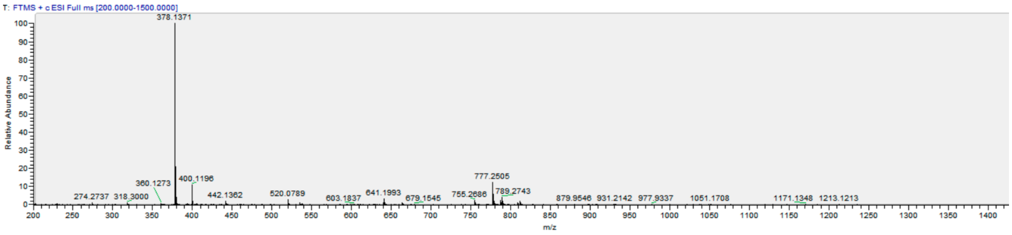

Figure S63 HR-ESI-MS spectrum of **7i**

HR-ESI-MS: m/z $[M+H]^+$ calcd for 378.1373 ($C_{20}H_{19}N_5OS$, found, 378.1371).

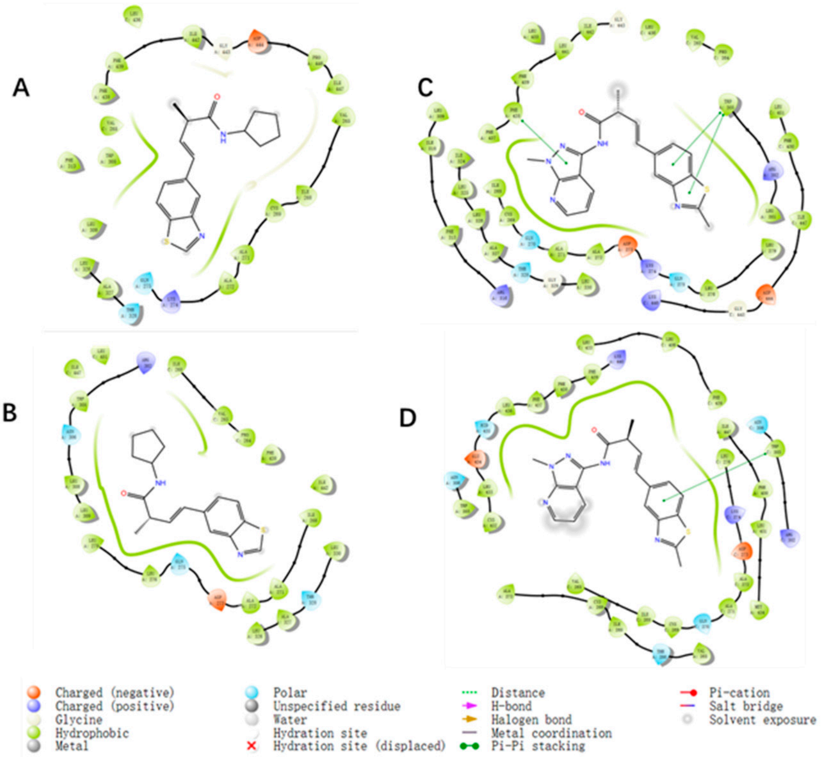

Figure S64. (A) 2D diagram of noncovalent interactions between S-**7b** and RXRα (B) 2D diagram of noncovalent interactions between R-**7b** and RXRα (C) 2D diagram of noncovalent interactions between S-**7i** and RXRα (D) 2D diagram of noncovalent interactions between R-**7i** and RXRα

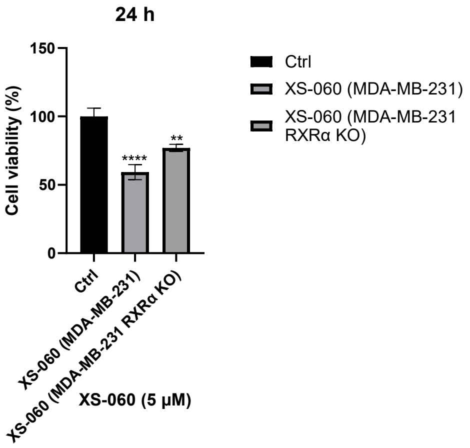

Figure S65. The cytotoxicity against MDA-MB-231 and MDA-MB-231 RXRα KO cells of positive control XS-060 at concentration of 5 μM. $p<0.01$ (**), $p<0.0001$ (****)
